# Supplementary material for: Evaluation of bioabsorbable calcium sulfate hemihydrate beads for local delivery of carboplatin
Source: PLoS One. 2020 Nov 5;15(11):e0241718. doi: 10.1371/journal.pone.0241718 (PMC7644016; doi:10.1371/journal.pone.0241718)
Supplement: S1 Statistics — (PDF) [file pone.0241718.s002.pdf]

The SAS System

The GLM Procedure

Class Level Information		
**Class**	**Levels**	**Values**
**Bead_Size**	2	3 5
**Dose**	4	20 50 100 500

**Number of Observations Read**	24
**Number of Observations Used**	24

The SAS System

The GLM Procedure

Dependent Variable: Hour Hour

Source	DF	Sum of Squares	Mean Square	F Value	Pr > F
Model	7	6362.625000	908.946429	8.60	0.0002
Error	16	1692.000000	105.750000		
Corrected Total	23	8054.625000			

R-Square	Coeff Var	Root MSE	Hour Mean
0.789934	25.78930	10.28348	39.87500

Source	DF	Type I SS	Mean Square	F Value	Pr > F
Bead_Size	1	570.375000	570.375000	5.39	0.0337
Dose	3	5773.125000	1924.375000	18.20	<.0001
Bead_Size*Dose	3	19.125000	6.375000	0.06	0.9799

Source	DF	Type III SS	Mean Square	F Value	Pr > F
Bead_Size	1	570.375000	570.375000	5.39	0.0337
Dose	3	5773.125000	1924.375000	18.20	<.0001
Bead_Size*Dose	3	19.125000	6.375000	0.06	0.9799

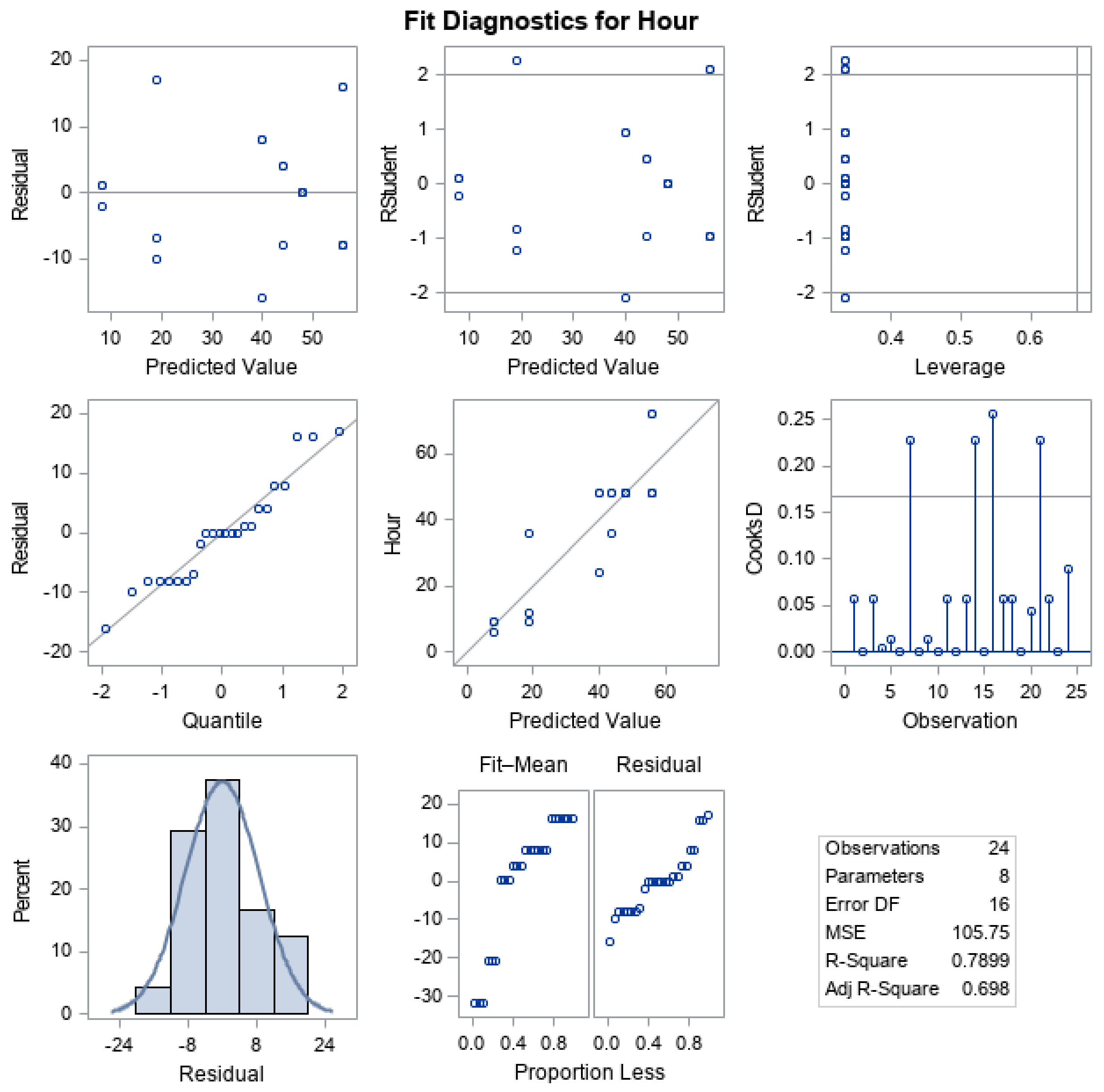

Observations	24
Parameters	8
Error DF	16
MSE	105.75
R-Square	0.7899
Adj R-Square	0.698

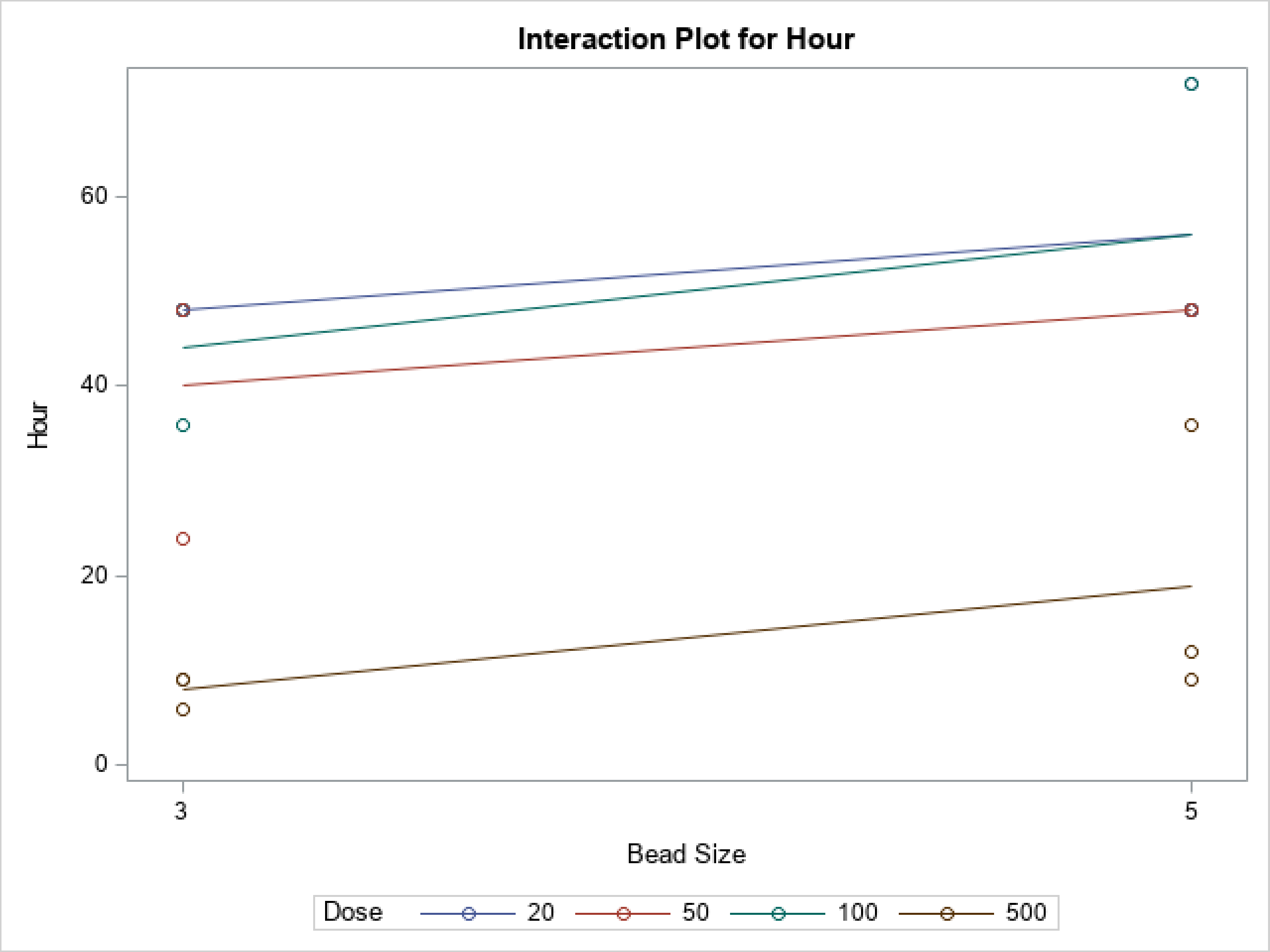
Interaction Plot for Hour
Hour
60
40
20
0
3
5
Bead Size
Dose
20
50
100
500

The SAS System

The GLM Procedure
Least Squares Means
Adjustment for Multiple Comparisons: Tukey

Bead_Size	Hour LSMEAN	Standard Error	H0:LSMEAN=0	H0:LSMean1=LSMean2
			Pr > |t|	Pr > |t|
3	35.0000000	2.9685855	<.0001	0.0337
5	44.7500000	2.9685855	<.0001	

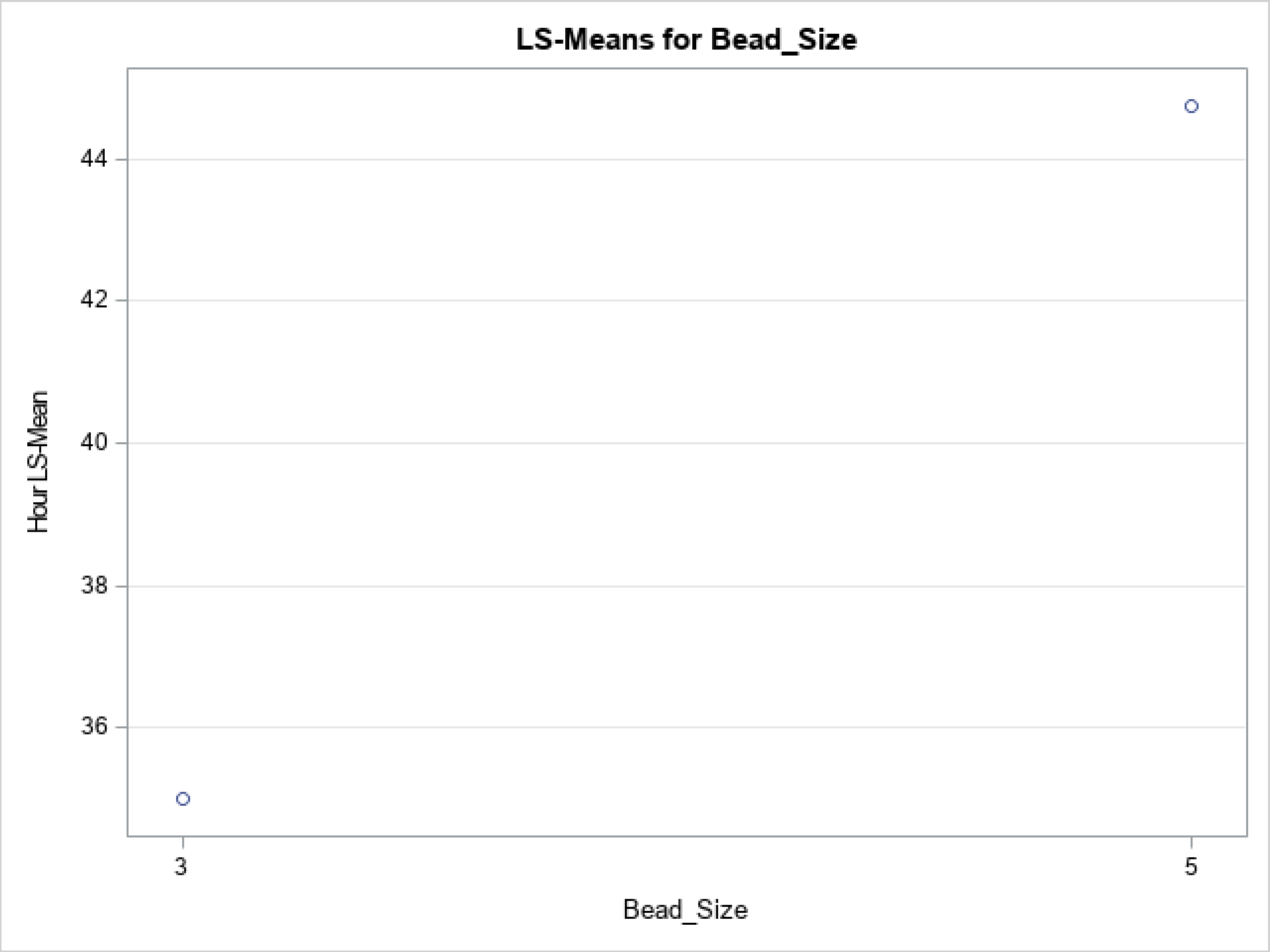

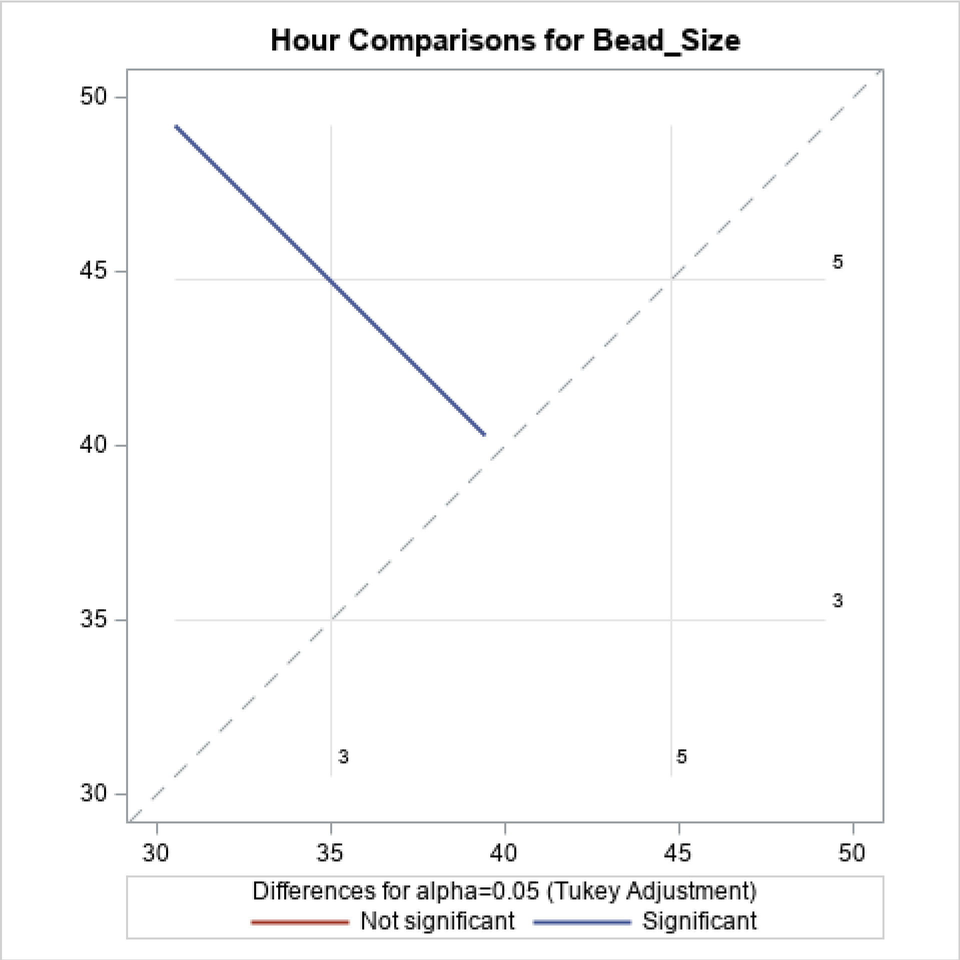
Hour Comparisons for Bead_Size
50
45
40
35
30
5
3
3
5
30
35
40
45
50
Differences for alpha=0.05 (Tukey Adjustment)
Not significant
Significant

The SAS System

The GLM Procedure
Least Squares Means
Adjustment for Multiple Comparisons: Tukey

Dose	Hour LSMEAN	Standard Error	Pr > |t|	LSMEAN Number
20	52.0000000	4.1982139	<.0001	1
50	44.0000000	4.1982139	<.0001	2
100	50.0000000	4.1982139	<.0001	3
500	13.5000000	4.1982139	0.0054	4

Least Squares Means for effect Dose
Pr > |t| for H0: LSMean(i)=LSMean(j)
Dependent Variable: Hour

i/j	1	2	3	4
1		0.5479	0.9863	<.0001
2	0.5479		0.7457	0.0005
3	0.9863	0.7457		<.0001
4	<.0001	0.0005	<.0001	

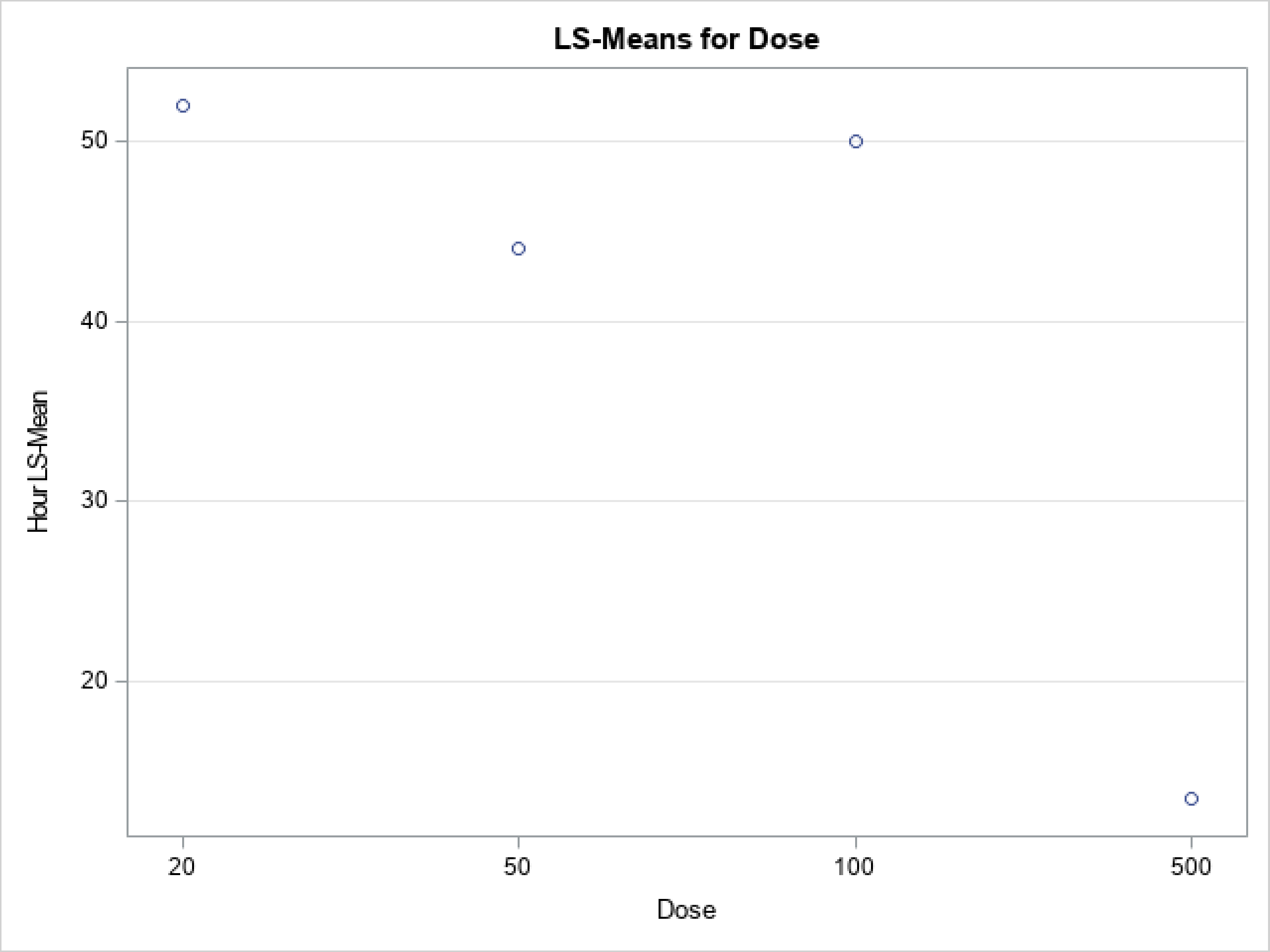
LS-Means for Dose
Hour LS-Mean
50
40
30
20
20
50
100
500
Dose

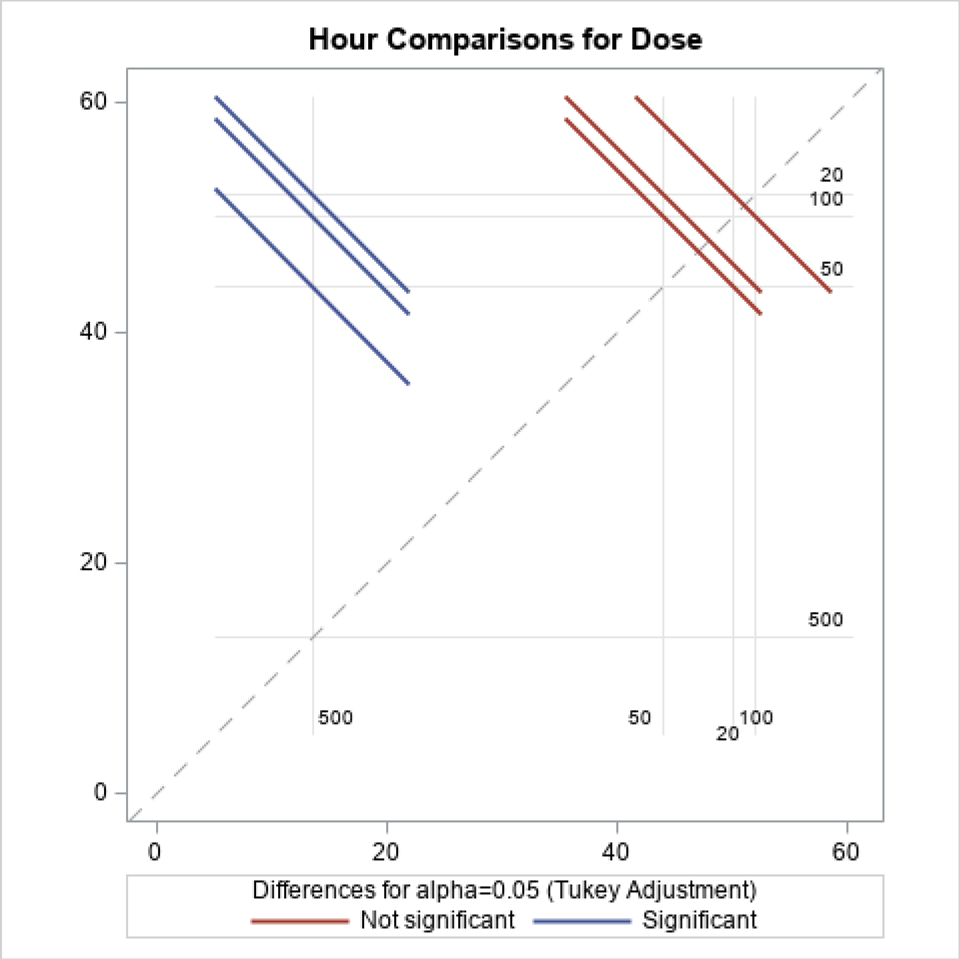
Hour Comparisons for Dose
60
40
20
0
0
20
40
60
20
100
50
500
500
50
20
100
Differences for alpha=0.05 (Tukey Adjustment)
Not significant
Significant

The SAS System

The GLM Procedure

Class Level Information		
**Class**	**Levels**	**Values**
**Bead_Size**	2	3 5
**Dose**	4	20 50 100 500

**Number of Observations Read**	24
**Number of Observations Used**	24

The SAS System

The GLM Procedure

Dependent Variable: Pct_Released

Source	DF	Sum of Squares	Mean Square	F Value	Pr > F
Model	4	0.26623608	0.06655902	6.60	0.0017
Error	19	0.19166594	0.01008768		
Corrected Total	23	0.45790202			

R-Square	Coeff Var	Root MSE	Pct_Released Mean
0.581426	16.21659	0.100437	0.619350

Source	DF	Type I SS	Mean Square	F Value	Pr > F
Bead_Size	1	0.00722454	0.00722454	0.72	0.4079
Dose	3	0.25901154	0.08633718	8.56	0.0008

Source	DF	Type III SS	Mean Square	F Value	Pr > F
Bead_Size	1	0.00722454	0.00722454	0.72	0.4079
Dose	3	0.25901154	0.08633718	8.56	0.0008

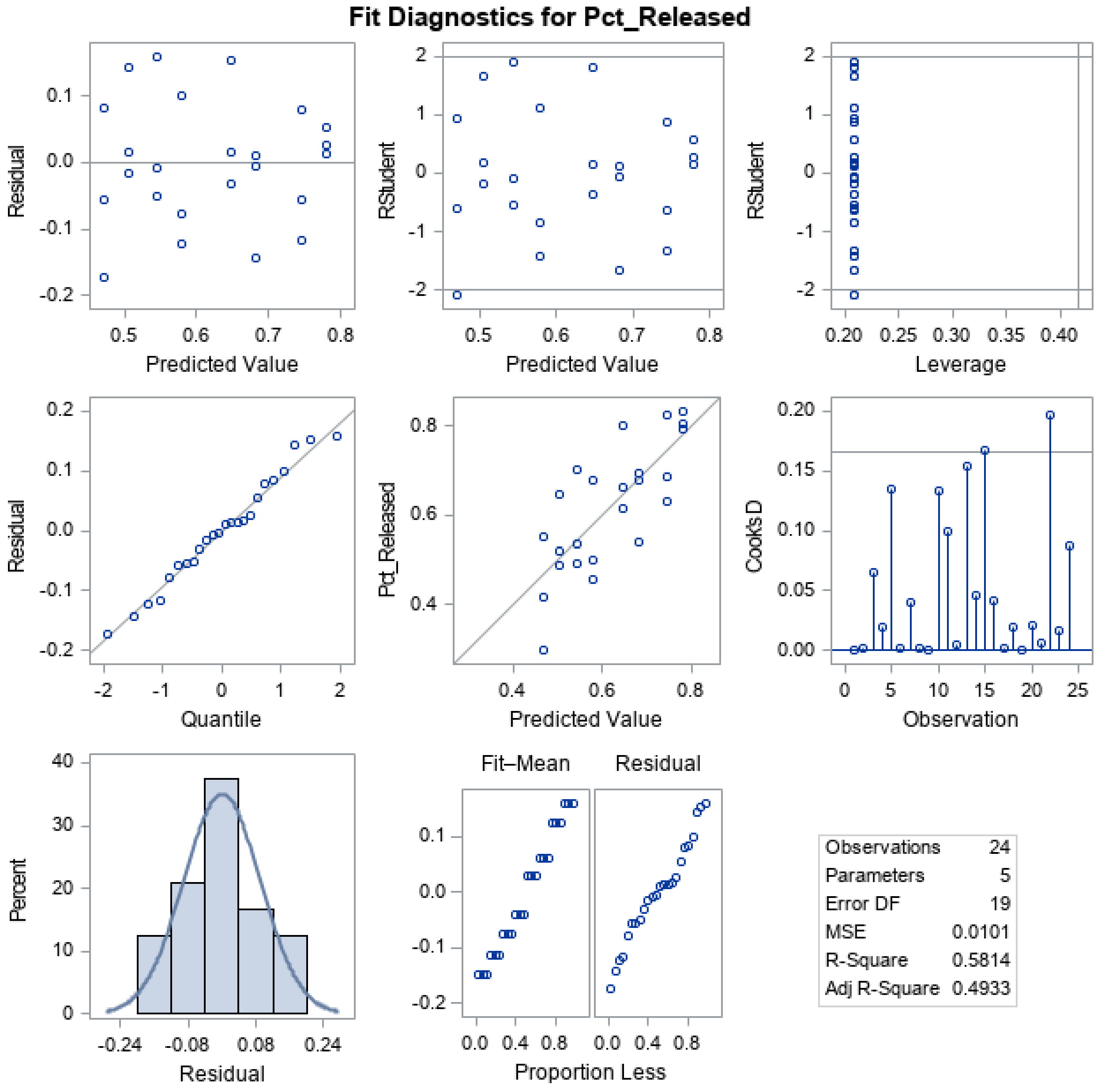

Observations	24
Parameters	5
Error DF	19
MSE	0.0101
R-Square	0.5814
Adj R-Square	0.4933

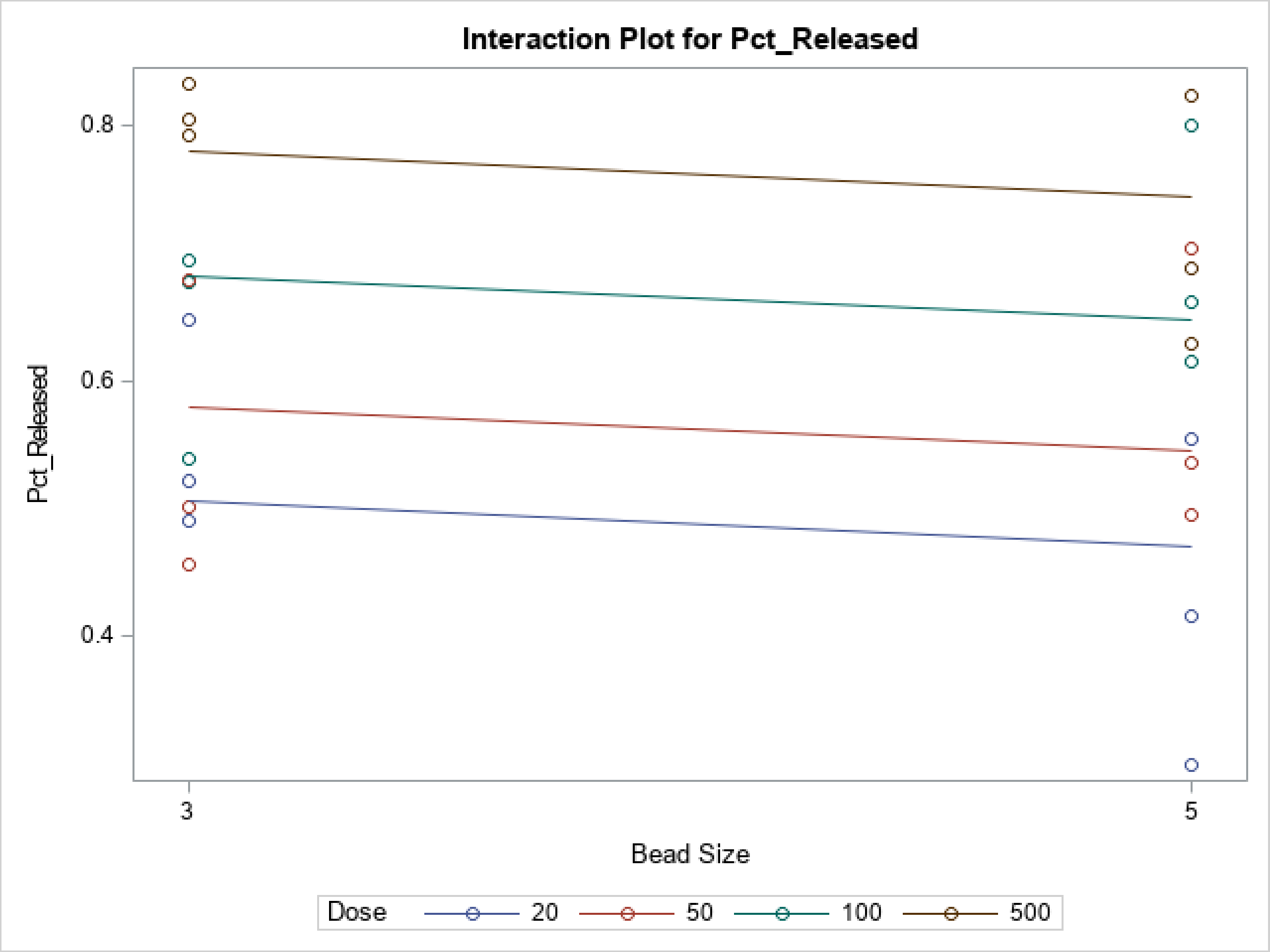
Interaction Plot for Pct_Released
0.8
0.6
0.4
Pct_Released
3
5
Bead Size
Dose
20
50
100
500

The SAS System

The GLM Procedure

Class Level Information		
**Class**	**Levels**	**Values**
**Bead_Size**	2	3 5
**Dose**	5	0 20 50 100 500

**Number of Observations Read**	30
**Number of Observations Used**	24

The SAS System

The GLM Procedure

Dependent Variable: Pct_Released

Source	DF	Sum of Squares	Mean Square	F Value	Pr > F
Model	4	0.08462077	0.02115519	1.66	0.2007
Error	19	0.24212989	0.01274368		
Corrected Total	23	0.32675066			

R-Square	Coeff Var	Root MSE	Pct_Released Mean
0.258977	20.54936	0.112888	0.549350

Source	DF	Type I SS	Mean Square	F Value	Pr > F
Bead_Size	1	0.00183750	0.00183750	0.14	0.7084
Dose	3	0.08278327	0.02759442	2.17	0.1256

Source	DF	Type III SS	Mean Square	F Value	Pr > F
Bead_Size	1	0.00183750	0.00183750	0.14	0.7084
Dose	3	0.08278327	0.02759442	2.17	0.1256

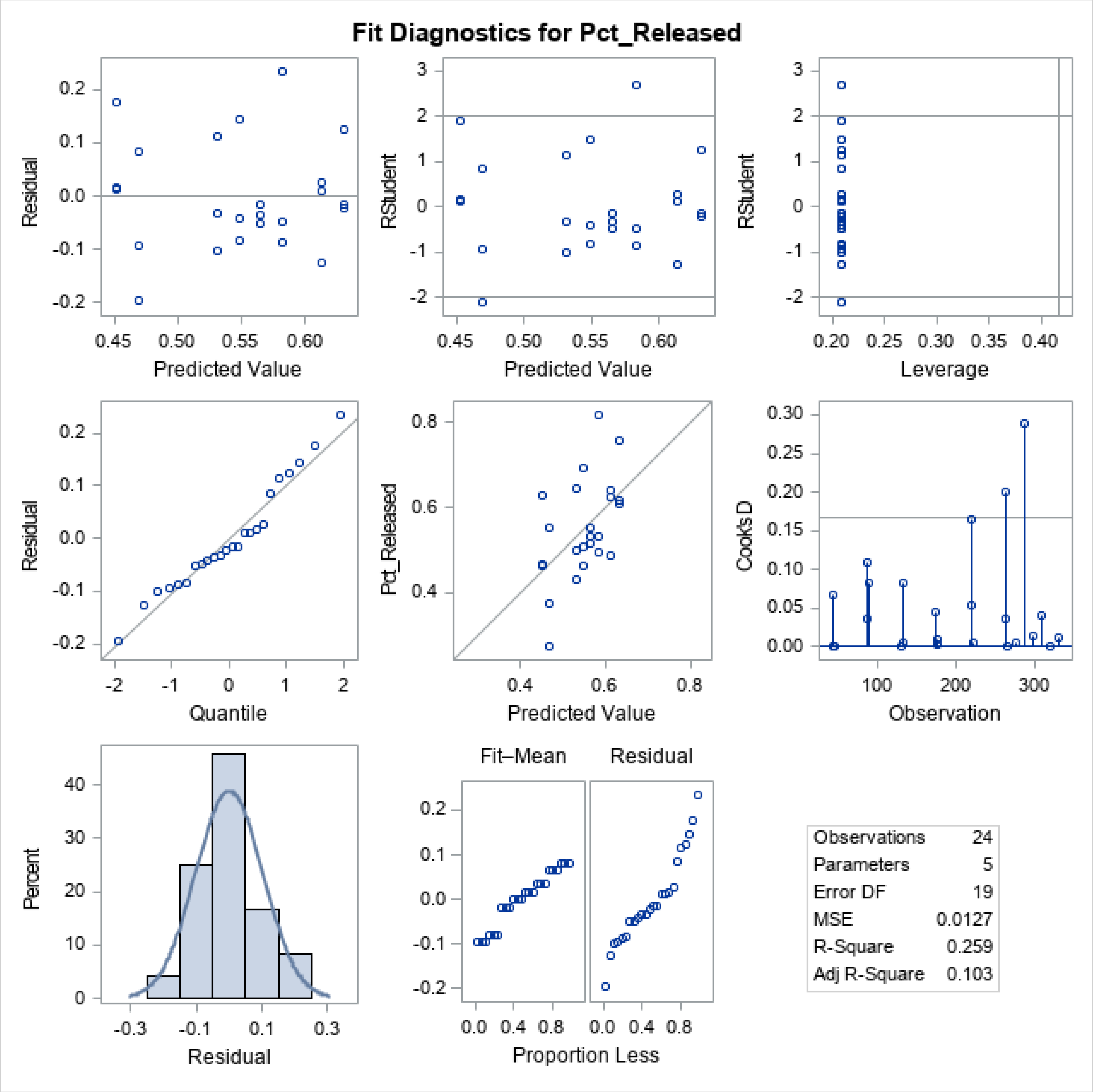
Fit Diagnostics for Pct_Released
Residual
Predicted Value
RStudent
Leverage
Quantile
Pct_Released
Cook's D
Observation
Percent
Residual
Fit–Mean
Proportion Less
Observations 24
Parameters 5
Error DF 19
MSE 0.0127
R-Square 0.259
Adj R-Square 0.103

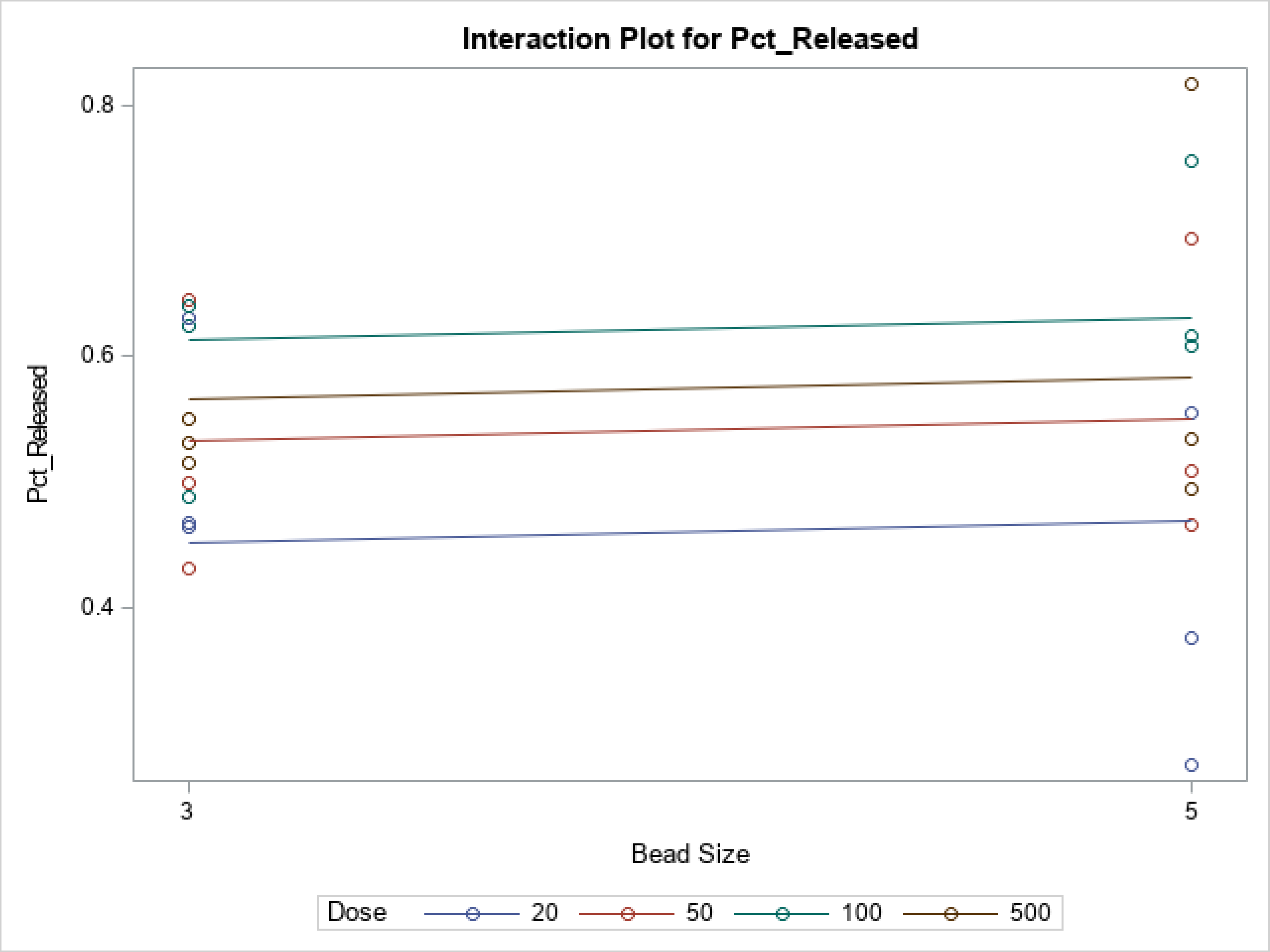
Interaction Plot for Pct_Released
0.8
0.6
0.4
Pct_Released
3
5
Bead Size
Dose
20
50
100
500

The SAS System

The GLM Procedure
Least Squares Means
Adjustment for Multiple Comparisons: Tukey

Dose	Pct_Released LSMEAN	Standard Error	Pr > |t|	LSMEAN Number
20	0.46100000	0.04608629	<.0001	1
50	0.54040000	0.04608629	<.0001	2
100	0.62226667	0.04608629	<.0001	3
500	0.57373333	0.04608629	<.0001	4

Least Squares Means for effect Dose
Pr > |t| for H0: LSMean(i)=LSMean(j)
Dependent Variable: Pct_Released

i/j	1	2	3	4
1		0.6233	0.0967	0.3365
2	0.6233		0.6005	0.9554
3	0.0967	0.6005		0.8778
4	0.3365	0.9554	0.8778	

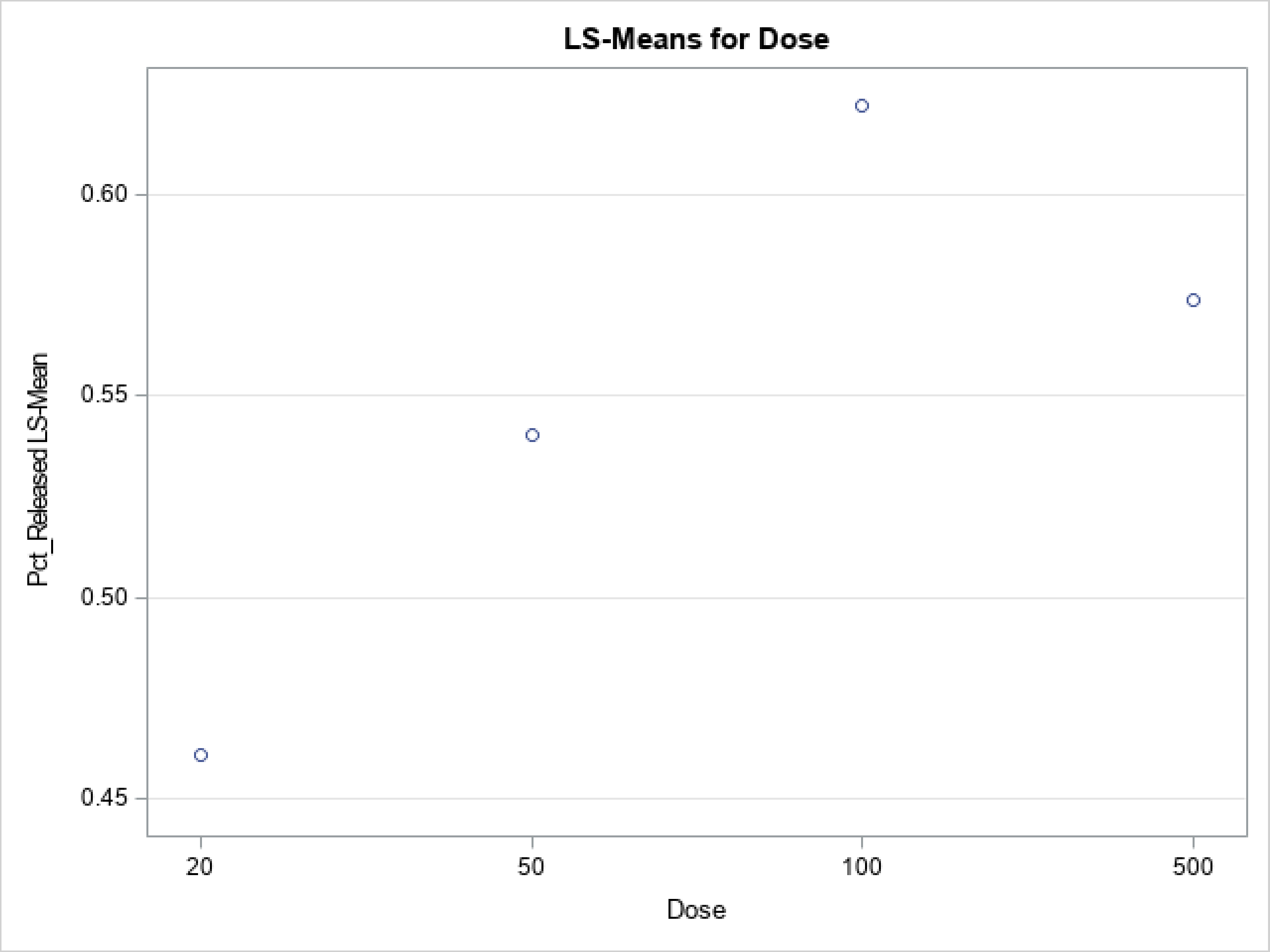
LS-Means for Dose
Pct_Released LS-Mean
0.45
0.50
0.55
0.60
20
50
100
500
Dose

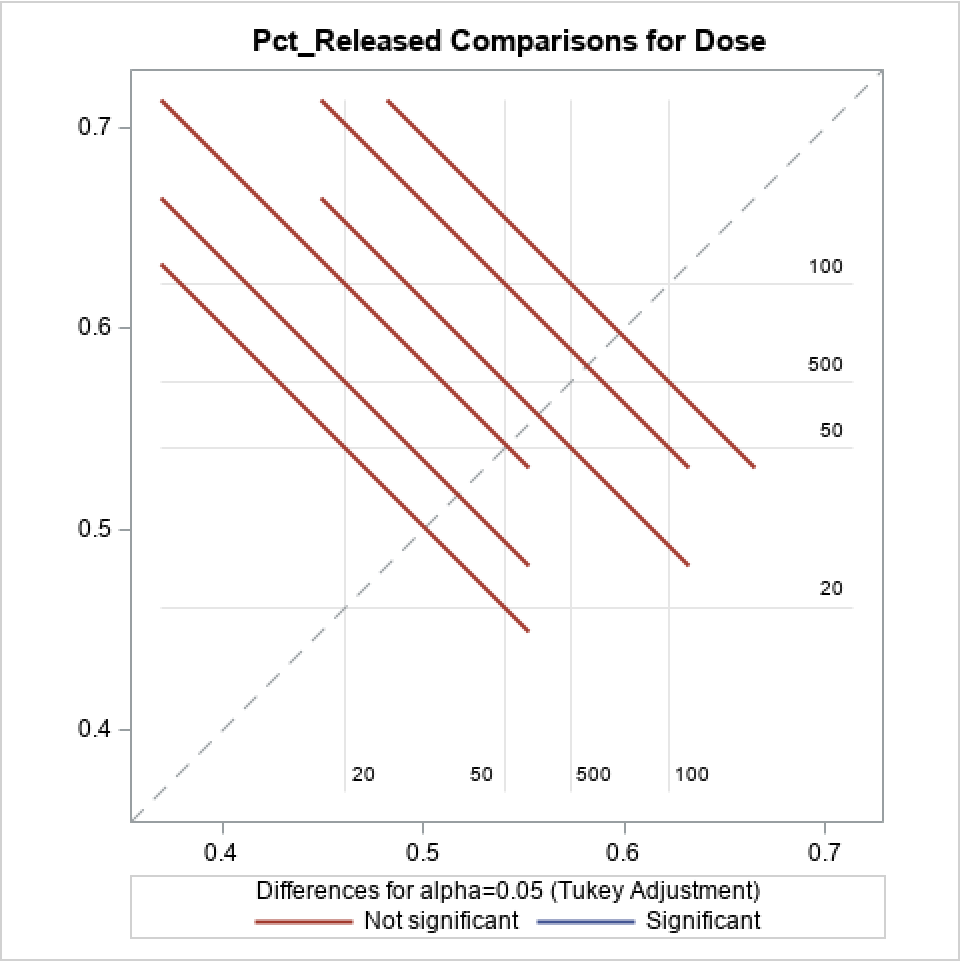

Pct_Released Comparisons for Dose
0.7
0.6
0.5
0.4
100
500
50
20
20
50
500
100
0.4
0.5
0.6
0.7
Differences for alpha=0.05 (Tukey Adjustment)
Not significant
Significant

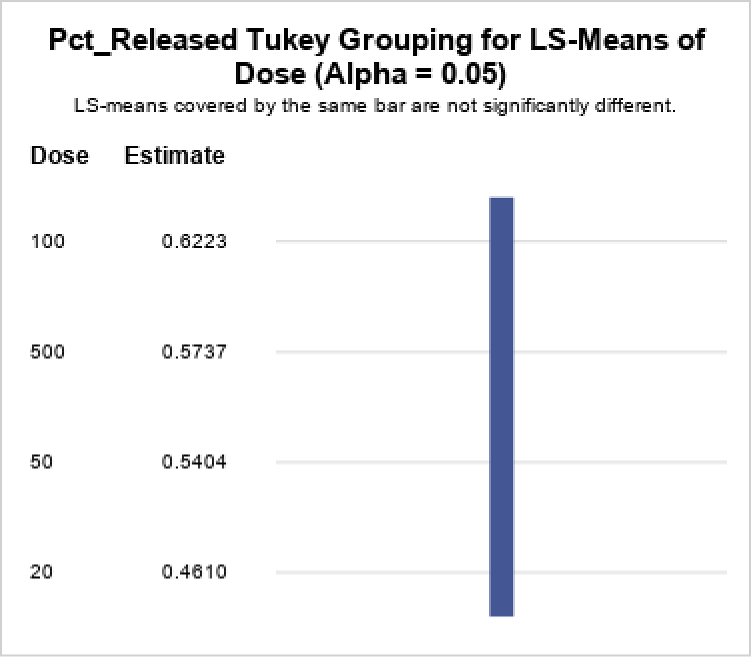

Pct_Released Tukey Grouping for LS-Means of Dose (Alpha = 0.05)
LS-means covered by the same bar are not significantly different.
Dose
Estimate
100
0.6223
500
0.5737
50
0.5404
20
0.4610

The SAS System

The GLM Procedure

Class Level Information		
Class	Levels	Values
Bead_Size	2	3 5
Dose	4	20 50 100 500

Number of Observations Read	24
Number of Observations Used	24

The SAS System

The GLM Procedure

Dependent Variable: logRateAdj

Source	DF	Sum of Squares	Mean Square	F Value	Pr > F
Model	4	15.39601578	3.84900394	34.15	<.0001
Error	19	2.14145282	0.11270804		
Corrected Total	23	17.53746860			

R-Square	Coeff Var	Root MSE	logRateAdj Mean
0.877893	-8.381695	0.335720	-4.005397

Source	DF	Type I SS	Mean Square	F Value	Pr > F
Bead_Size	1	0.92648034	0.92648034	8.22	0.0099
Dose	3	14.46953544	4.82317848	42.79	<.0001

Source	DF	Type III SS	Mean Square	F Value	Pr > F
Bead_Size	1	0.92648034	0.92648034	8.22	0.0099
Dose	3	14.46953544	4.82317848	42.79	<.0001

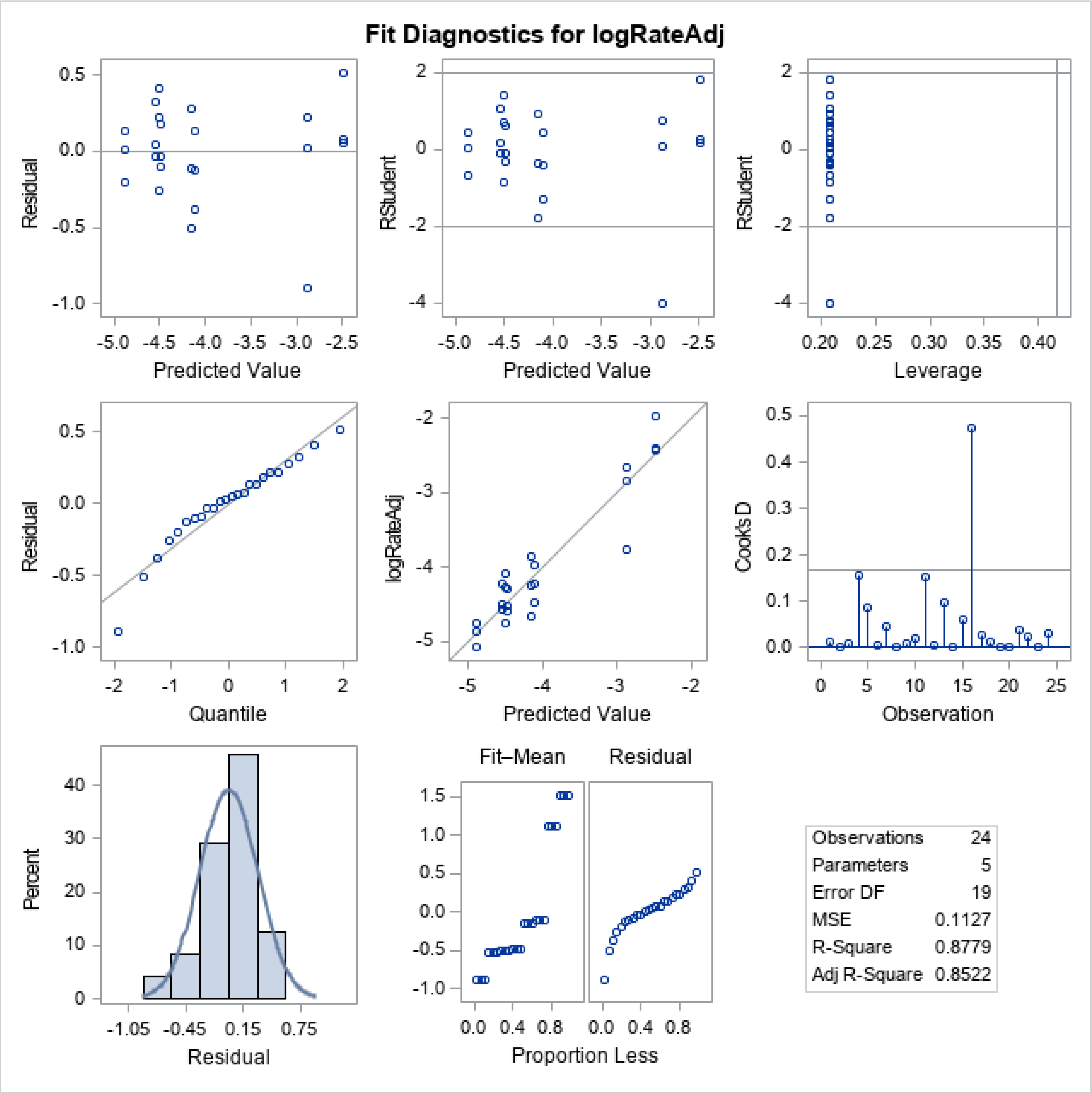
Fit Diagnostics for logRateAdj
Residual
RStudent
Predicted Value
Leverage
Quantile
logRateAdj
Cook's D
Observation
Percent
Residual
Fit–Mean
Residual
Proportion Less
Observations 24
Parameters 5
Error DF 19
MSE 0.1127
R-Square 0.8779
Adj R-Square 0.8522

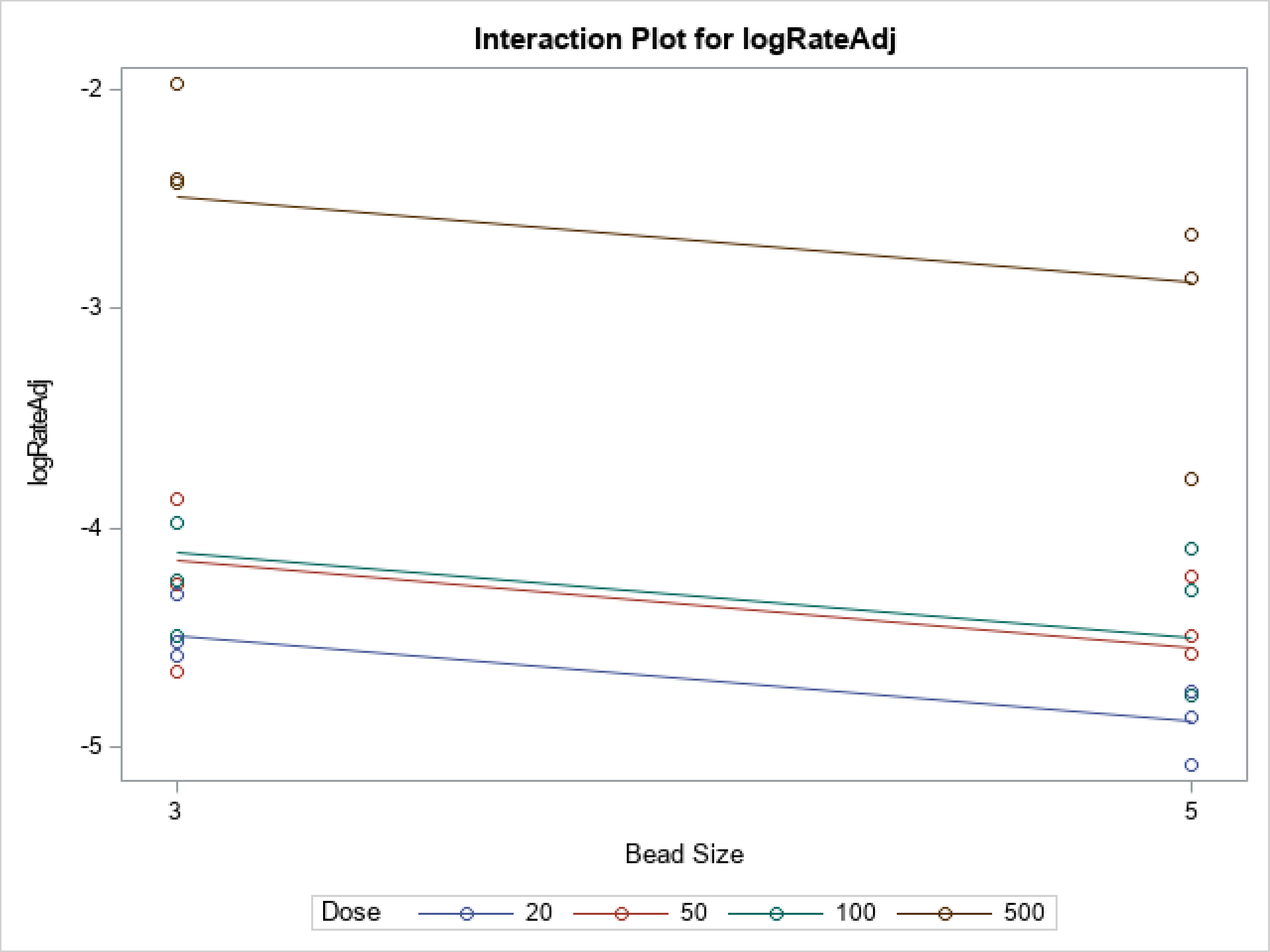
Interaction Plot for logRateAdj
logRateAdj
-2
-3
-4
-5
3
5
Bead Size
Dose
20
50
100
500

The SAS System

The GLM Procedure
Least Squares Means
Adjustment for Multiple Comparisons: Tukey

Dose	logRateAdj LSMEAN	Standard Error	Pr > |t|	LSMEAN Number
20	-4.68471596	0.13705719	<.0001	1
50	-4.34585362	0.13705719	<.0001	2
100	-4.30613775	0.13705719	<.0001	3
500	-2.68488222	0.13705719	<.0001	4

Least Squares Means for effect Dose
Pr > |t| for H0: LSMean(i)=LSMean(j)
Dependent Variable: logRateAdj

i/j	1	2	3	4
1		0.3277	0.2402	<.0001
2	0.3277		0.9968	<.0001
3	0.2402	0.9968		<.0001
4	<.0001	<.0001	<.0001	

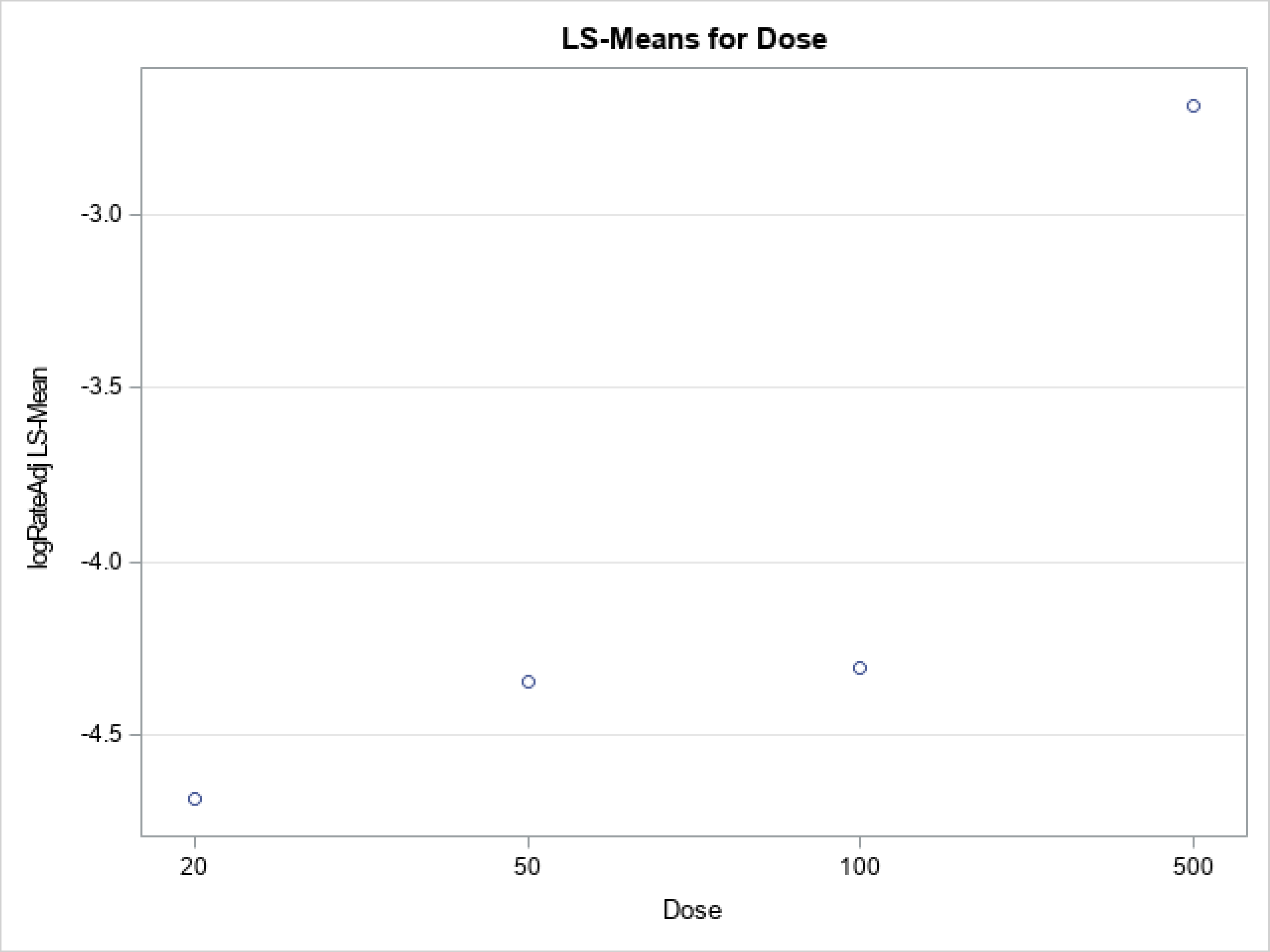
LS-Means for Dose
logRateAdj LS-Mean
-3.0
-3.5
-4.0
-4.5
20
50
100
500
Dose

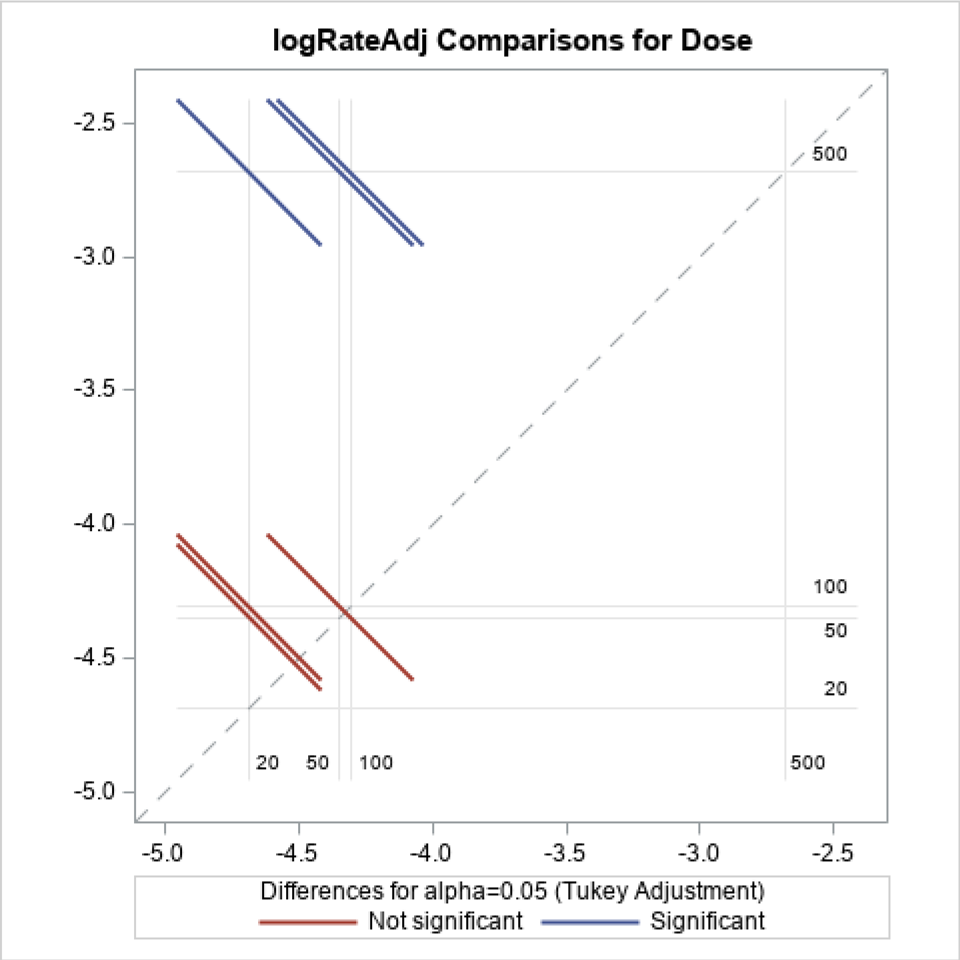
logRateAdj Comparisons for Dose
-2.5
-3.0
-3.5
-4.0
-4.5
-5.0
500
100
50
20
20 50 100
500
-5.0 -4.5 -4.0 -3.5 -3.0 -2.5
Differences for alpha=0.05 (Tukey Adjustment)
Not significant
Significant

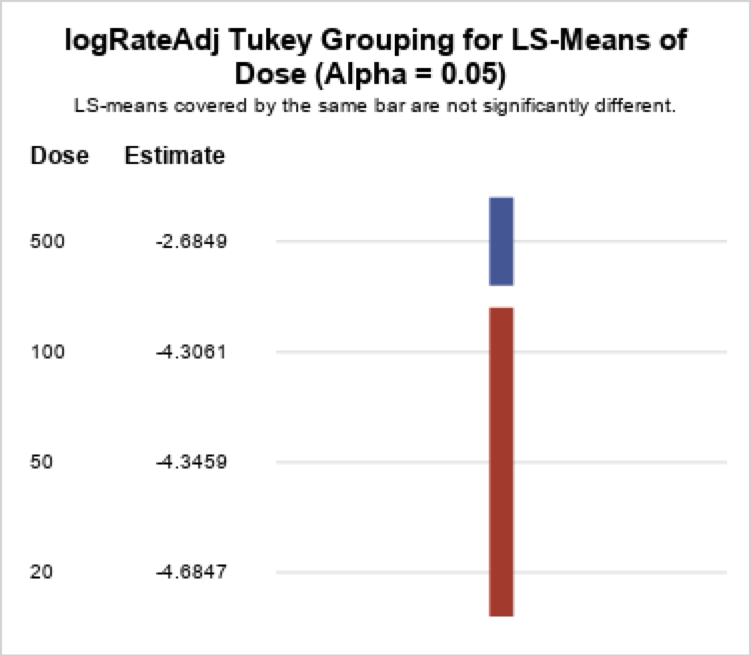
logRateAdj Tukey Grouping for LS-Means of Dose (Alpha = 0.05)
LS-means covered by the same bar are not significantly different.
Dose Estimate
500 -2.6849
100 -4.3061
50 -4.3459
20 -4.6847

The SAS System

The GLM Procedure
Least Squares Means
Adjustment for Multiple Comparisons: Tukey

Bead_Size	logRateAdj LSMEAN	Standard Error	H0:LSMEAN=0	H0:LSMean1=LSMean2
			Pr > |t|	Pr > |t|
3	-3.80892004	0.09691407	<.0001	0.0099
5	-4.20187473	0.09691407	<.0001	

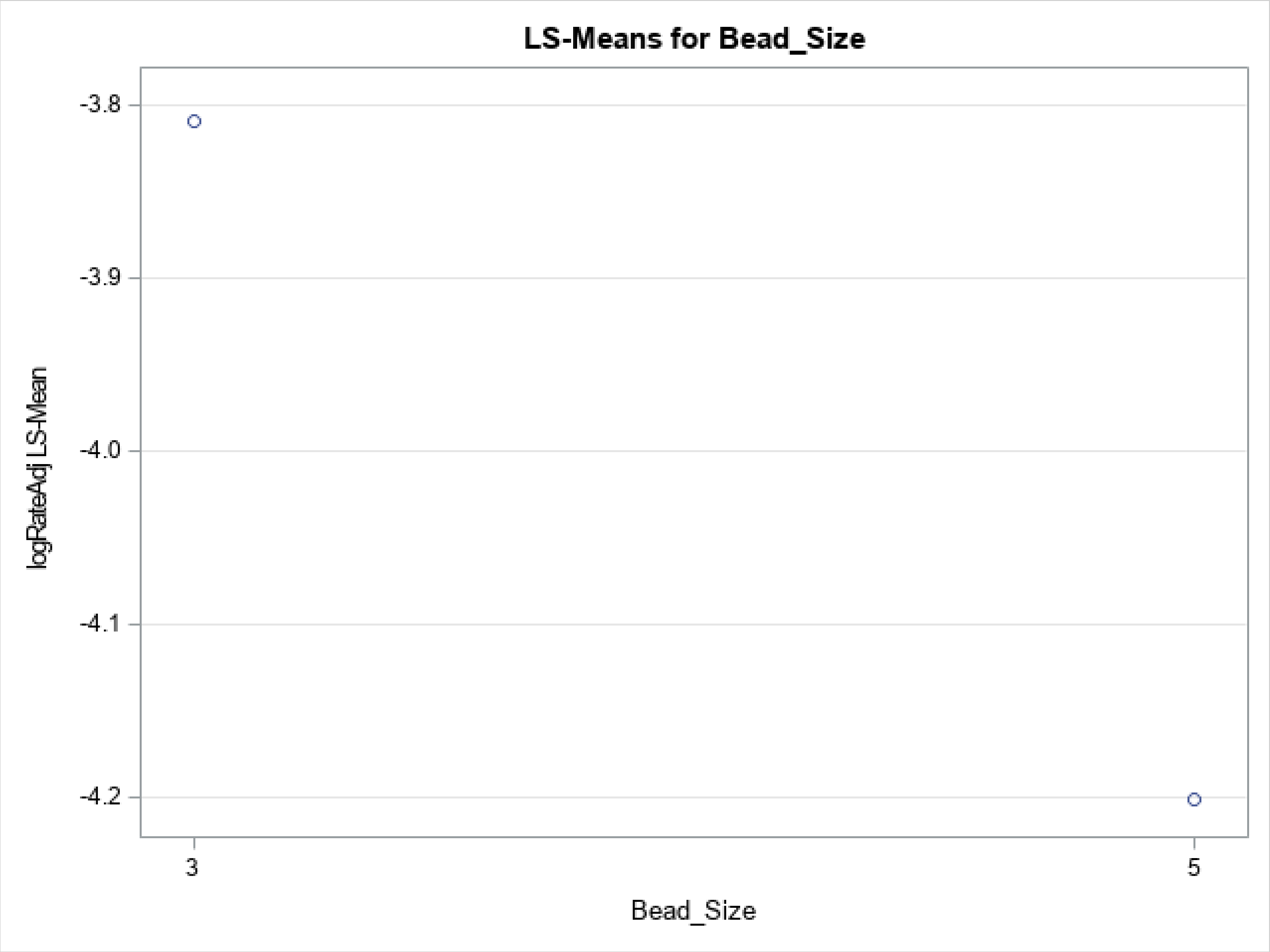

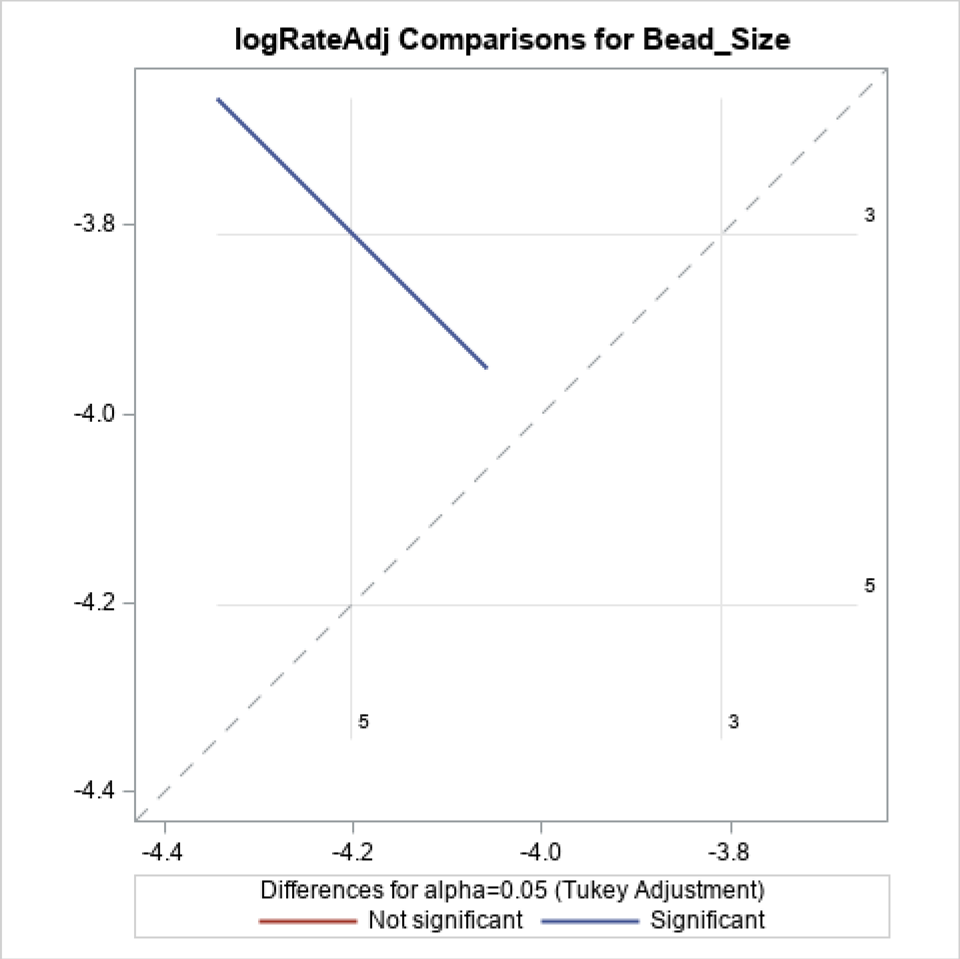
logRateAdj Comparisons for Bead_Size
-3.8
-4.0
-4.2
-4.4
3
5
5
3
-4.4
-4.2
-4.0
-3.8
Differences for alpha=0.05 (Tukey Adjustment)
Not significant
Significant

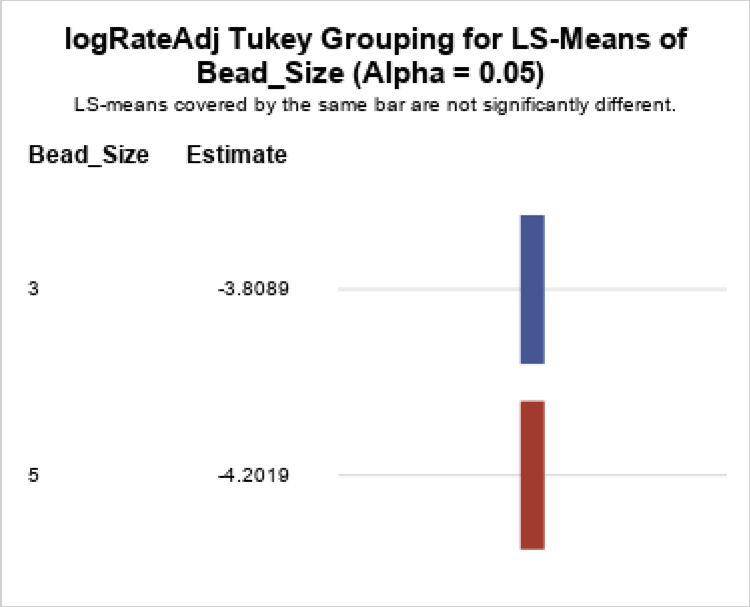
logRateAdj Tukey Grouping for LS-Means of Bead_Size (Alpha = 0.05)
LS-means covered by the same bar are not significantly different.
Bead_Size
Estimate
3
-3.8089
5
-4.2019

The SAS System

The Mixed Procedure

Model Information	
Data Set	WORK.POWDERSTUDY3
Dependent Variable	Pct_Released
Covariance Structure	Unstructured
Subject Effect	SubjectID
Estimation Method	REML
Residual Variance Method	None
Fixed Effects SE Method	Model-Based
Degrees of Freedom Method	Between-Within

Class Level Information		
Class	Levels	Values
Hour	10	1 2 3 6 9 12 24 36 48 72
Group	3	A B C
Bead_Size	2	3 5
Dose	4	20 50 100 500
SubjectID	24	3A100 3A20 3A50 3A500 3B100 3B20 3B50 3B500 3C100 3C20 3C50 3C500 5A100 5A20 5A50 5A500 5B100 5B20 5B50 5B500 5C100 5C20 5C50 5C500

Dimensions	
Covariance Parameters	55
Columns in X	85
Columns in Z	0
Subjects	24
Max Obs per Subject	10

Number of Observations	
Number of Observations Read	240
Number of Observations Used	240
Number of Observations Not Used	0

Iteration History			
Iteration	Evaluations	-2 Res Log Like	Criterion

**0**	1	-241.62568486	
**1**	2	-478.32196849	0.00118155
**2**	1	-478.88598927	0.00011503
**3**	1	-478.93702650	0.00000215
**4**	1	-478.93792623	0.00000000

Convergence criteria met.

Covariance Parameter Estimates		
**Cov Parm**	**Subject**	**Estimate**
**UN(1,1)**	SubjectID	0.006528
**UN(2,1)**	SubjectID	0.000894
**UN(2,2)**	SubjectID	0.009583
**UN(3,1)**	SubjectID	0.004329
**UN(3,2)**	SubjectID	0.005909
**UN(3,3)**	SubjectID	0.009972
**UN(4,1)**	SubjectID	0.001461
**UN(4,2)**	SubjectID	0.004169
**UN(4,3)**	SubjectID	0.001567
**UN(4,4)**	SubjectID	0.008384
**UN(5,1)**	SubjectID	0.002110
**UN(5,2)**	SubjectID	0.001167
**UN(5,3)**	SubjectID	0.000596
**UN(5,4)**	SubjectID	0.004946
**UN(5,5)**	SubjectID	0.006808
**UN(6,1)**	SubjectID	0.001781
**UN(6,2)**	SubjectID	0.003200
**UN(6,3)**	SubjectID	0.002894
**UN(6,4)**	SubjectID	0.004425
**UN(6,5)**	SubjectID	0.005346
**UN(6,6)**	SubjectID	0.008463
**UN(7,1)**	SubjectID	0.000894
**UN(7,2)**	SubjectID	0.002217
**UN(7,3)**	SubjectID	0.001692
**UN(7,4)**	SubjectID	0.003115

**UN(7,5)**	SubjectID	0.004086
**UN(7,6)**	SubjectID	0.008282
**UN(7,7)**	SubjectID	0.01102
**UN(8,1)**	SubjectID	0.002598
**UN(8,2)**	SubjectID	0.004319
**UN(8,3)**	SubjectID	0.004756
**UN(8,4)**	SubjectID	0.002076
**UN(8,5)**	SubjectID	0.002654
**UN(8,6)**	SubjectID	0.008296
**UN(8,7)**	SubjectID	0.01055
**UN(8,8)**	SubjectID	0.01462
**UN(9,1)**	SubjectID	0.001817
**UN(9,2)**	SubjectID	0.003220
**UN(9,3)**	SubjectID	0.003761
**UN(9,4)**	SubjectID	0.001615
**UN(9,5)**	SubjectID	0.003522
**UN(9,6)**	SubjectID	0.008687
**UN(9,7)**	SubjectID	0.01024
**UN(9,8)**	SubjectID	0.01356
**UN(9,9)**	SubjectID	0.01457
**UN(10,1)**	SubjectID	0.002367
**UN(10,2)**	SubjectID	0.003021
**UN(10,3)**	SubjectID	0.004261
**UN(10,4)**	SubjectID	0.002013
**UN(10,5)**	SubjectID	0.003666
**UN(10,6)**	SubjectID	0.008730
**UN(10,7)**	SubjectID	0.01024
**UN(10,8)**	SubjectID	0.01381
**UN(10,9)**	SubjectID	0.01462
**UN(10,10)**	SubjectID	0.01507

**Fit Statistics**	
**-2 Res Log Likelihood**	-478.9
**AIC (Smaller is Better)**	-368.9
**AICC (Smaller is Better)**	-321.9

BIC (Smaller is Better)	-304.1

Null Model Likelihood Ratio Test		
DF	Chi-Square	Pr > ChiSq
54	237.31	<.0001

Information Criteria						
Neg2LogLike	Parms	AIC	AICC	HQIC	BIC	CAIC
-478.9	55	-368.9	-321.9	-351.7	-304.1	-249.1

Type 3 Tests of Fixed Effects				
Effect	Num DF	Den DF	F Value	Pr > F
Bead_Size	1	16	1.45	0.2455
Dose	3	16	6.01	0.0061
Bead_Size*Dose	3	16	6.09	0.0058
Hour	9	16	101.05	<.0001
Hour*Bead_Size	9	16	4.78	0.0033
Hour*Dose	27	16	8.51	<.0001

Least Squares Means								
Effect	Hour	Bead Size	Dose	Estimate	Standard Error	DF	t Value	Pr > |t|
Hour*Bead_Size	1	3		0.1550	0.02332	16	6.65	<.0001
Hour*Bead_Size	1	5		0.1345	0.02332	16	5.77	<.0001
Hour*Bead_Size	2	3		0.2766	0.02826	16	9.79	<.0001
Hour*Bead_Size	2	5		0.2450	0.02826	16	8.67	<.0001
Hour*Bead_Size	3	3		0.3527	0.02883	16	12.24	<.0001
Hour*Bead_Size	3	5		0.2989	0.02883	16	10.37	<.0001
Hour*Bead_Size	6	3		0.4866	0.02643	16	18.41	<.0001
Hour*Bead_Size	6	5		0.3612	0.02643	16	13.67	<.0001
Hour*Bead_Size	9	3		0.5118	0.02382	16	21.49	<.0001
Hour*Bead_Size	9	5		0.4587	0.02382	16	19.26	<.0001
Hour*Bead_Size	12	3		0.5333	0.02656	16	20.08	<.0001
Hour*Bead_Size	12	5		0.5092	0.02656	16	19.18	<.0001
Hour*Bead_Size	24	3		0.5550	0.03030	16	18.32	<.0001
Hour*Bead_Size	24	5		0.5041	0.03030	16	16.64	<.0001
Hour*Bead_Size	36	3		0.5525	0.03491	16	15.83	<.0001

**Hour*Bead_Size**	36	5		0.5352	0.03491	16	15.33	<.0001
**Hour*Bead_Size**	48	3		0.5685	0.03484	16	16.32	<.0001
**Hour*Bead_Size**	48	5		0.5714	0.03484	16	16.40	<.0001
**Hour*Bead_Size**	72	3		0.5406	0.03544	16	15.25	<.0001
**Hour*Bead_Size**	72	5		0.5581	0.03544	16	15.75	<.0001
**Hour*Dose**	1		20	0.1427	0.03298	16	4.33	0.0005
**Hour*Dose**	1		50	0.2387	0.03298	16	7.24	<.0001
**Hour*Dose**	1		100	0.08500	0.03298	16	2.58	0.0203
**Hour*Dose**	1		500	0.1127	0.03298	16	3.42	0.0035
**Hour*Dose**	2		20	0.1940	0.03996	16	4.85	0.0002
**Hour*Dose**	2		50	0.3049	0.03996	16	7.63	<.0001
**Hour*Dose**	2		100	0.1711	0.03996	16	4.28	0.0006
**Hour*Dose**	2		500	0.3732	0.03996	16	9.34	<.0001
**Hour*Dose**	3		20	0.2680	0.04077	16	6.57	<.0001
**Hour*Dose**	3		50	0.3527	0.04077	16	8.65	<.0001
**Hour*Dose**	3		100	0.2675	0.04077	16	6.56	<.0001
**Hour*Dose**	3		500	0.4152	0.04077	16	10.18	<.0001
**Hour*Dose**	6		20	0.3200	0.03738	16	8.56	<.0001
**Hour*Dose**	6		50	0.3853	0.03738	16	10.31	<.0001
**Hour*Dose**	6		100	0.3686	0.03738	16	9.86	<.0001
**Hour*Dose**	6		500	0.6217	0.03738	16	16.63	<.0001
**Hour*Dose**	9		20	0.3613	0.03369	16	10.73	<.0001
**Hour*Dose**	9		50	0.4524	0.03369	16	13.43	<.0001
**Hour*Dose**	9		100	0.4349	0.03369	16	12.91	<.0001
**Hour*Dose**	9		500	0.6925	0.03369	16	20.56	<.0001
**Hour*Dose**	12		20	0.3920	0.03756	16	10.44	<.0001
**Hour*Dose**	12		50	0.4737	0.03756	16	12.61	<.0001
**Hour*Dose**	12		100	0.4962	0.03756	16	13.21	<.0001
**Hour*Dose**	12		500	0.7231	0.03756	16	19.25	<.0001
**Hour*Dose**	24		20	0.4277	0.04285	16	9.98	<.0001
**Hour*Dose**	24		50	0.5232	0.04285	16	12.21	<.0001
**Hour*Dose**	24		100	0.5549	0.04285	16	12.95	<.0001
**Hour*Dose**	24		500	0.6124	0.04285	16	14.29	<.0001
**Hour*Dose**	36		20	0.4550	0.04937	16	9.22	<.0001
**Hour*Dose**	36		50	0.5195	0.04937	16	10.52	<.0001

	36		100	0.6188	0.04937	16	12.53	<.0001
Hour*Dose	36		500	0.5821	0.04937	16	11.79	<.0001
Hour*Dose	48		20	0.4873	0.04927	16	9.89	<.0001
Hour*Dose	48		50	0.5601	0.04927	16	11.37	<.0001
Hour*Dose	48		100	0.6599	0.04927	16	13.39	<.0001
Hour*Dose	48		500	0.5725	0.04927	16	11.62	<.0001
Hour*Dose	72		20	0.4610	0.05012	16	9.20	<.0001
Hour*Dose	72		50	0.5404	0.05012	16	10.78	<.0001
Hour*Dose	72		100	0.6223	0.05012	16	12.42	<.0001
Hour*Dose	72		500	0.5737	0.05012	16	11.45	<.0001

Tests of Effect Slices

Effect	Hour	Num DF	Den DF	F Value	Pr > F
Hour*Bead_Size	1	1	16	0.39	0.5417
Hour*Bead_Size	2	1	16	0.62	0.4416
Hour*Bead_Size	3	1	16	1.74	0.2055
Hour*Bead_Size	6	1	16	11.26	0.0040
Hour*Bead_Size	9	1	16	2.48	0.1345
Hour*Bead_Size	12	1	16	0.41	0.5313
Hour*Bead_Size	24	1	16	1.41	0.2519
Hour*Bead_Size	36	1	16	0.12	0.7306
Hour*Bead_Size	48	1	16	0.00	0.9538
Hour*Bead_Size	72	1	16	0.12	0.7315
Hour*Dose	1	3	16	4.11	0.0243
Hour*Dose	2	3	16	5.65	0.0077
Hour*Dose	3	3	16	3.10	0.0564
Hour*Dose	6	3	16	13.00	0.0001
Hour*Dose	9	3	16	18.20	<.0001
Hour*Dose	12	3	16	14.26	<.0001
Hour*Dose	24	3	16	3.25	0.0493
Hour*Dose	36	3	16	2.13	0.1366
Hour*Dose	48	3	16	2.06	0.1456
Hour*Dose	72	3	16	1.83	0.1822

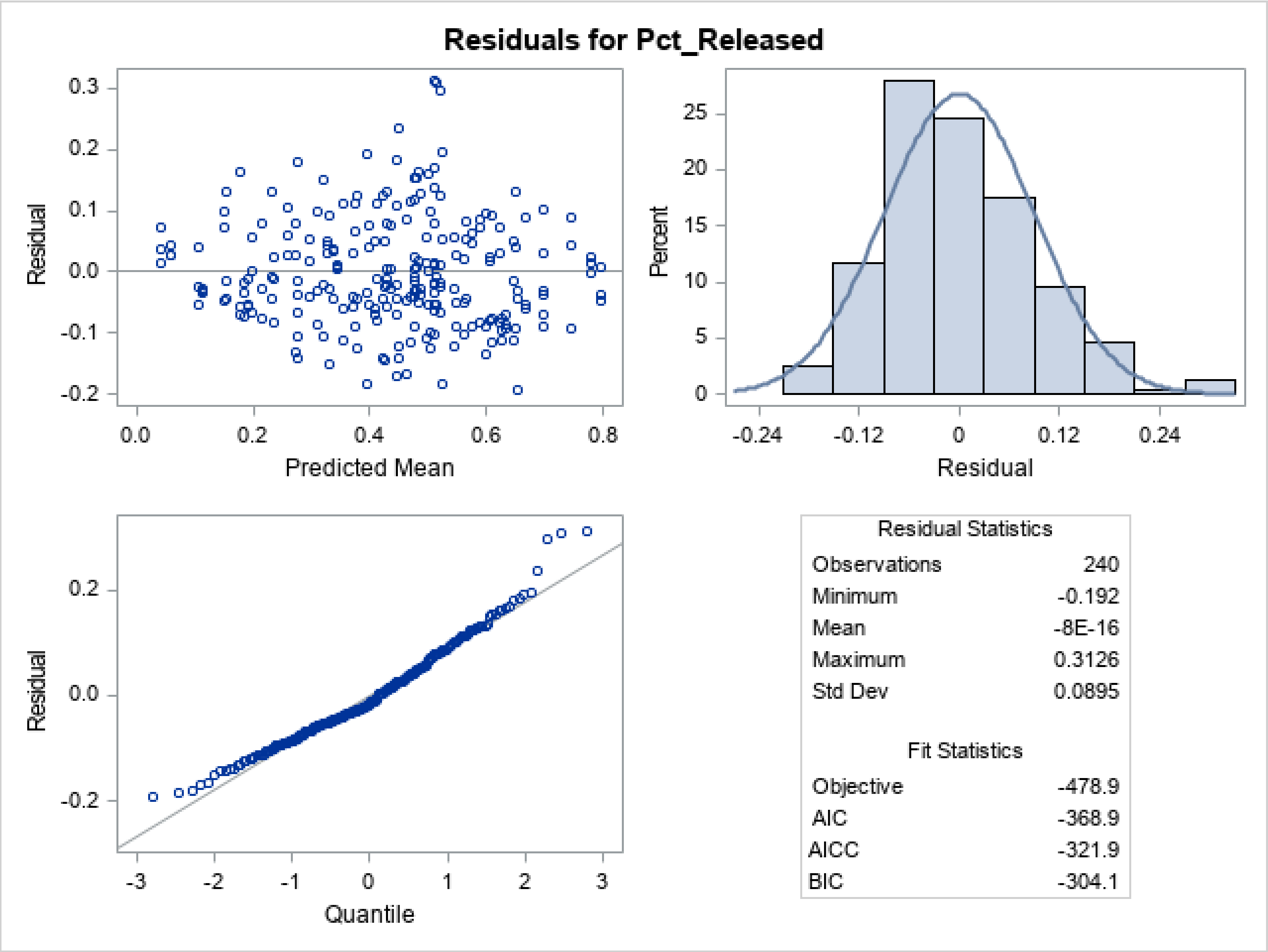
Residuals for Pct_Released
Residual
Predicted Mean
0.3
0.2
0.1
0.0
-0.1
-0.2
0.0
0.2
0.4
0.6
0.8
Percent
Residual
25
20
15
10
5
0
-0.24
-0.12
0
0.12
0.24
Residual
Quantile
-3
-2
-1
0
1
2
3
Residual Statistics
Observations 240
Minimum -0.192
Mean -8E-16
Maximum 0.3126
Std Dev 0.0895
Fit Statistics
Objective -478.9
AIC -368.9
AICC -321.9
BIC -304.1

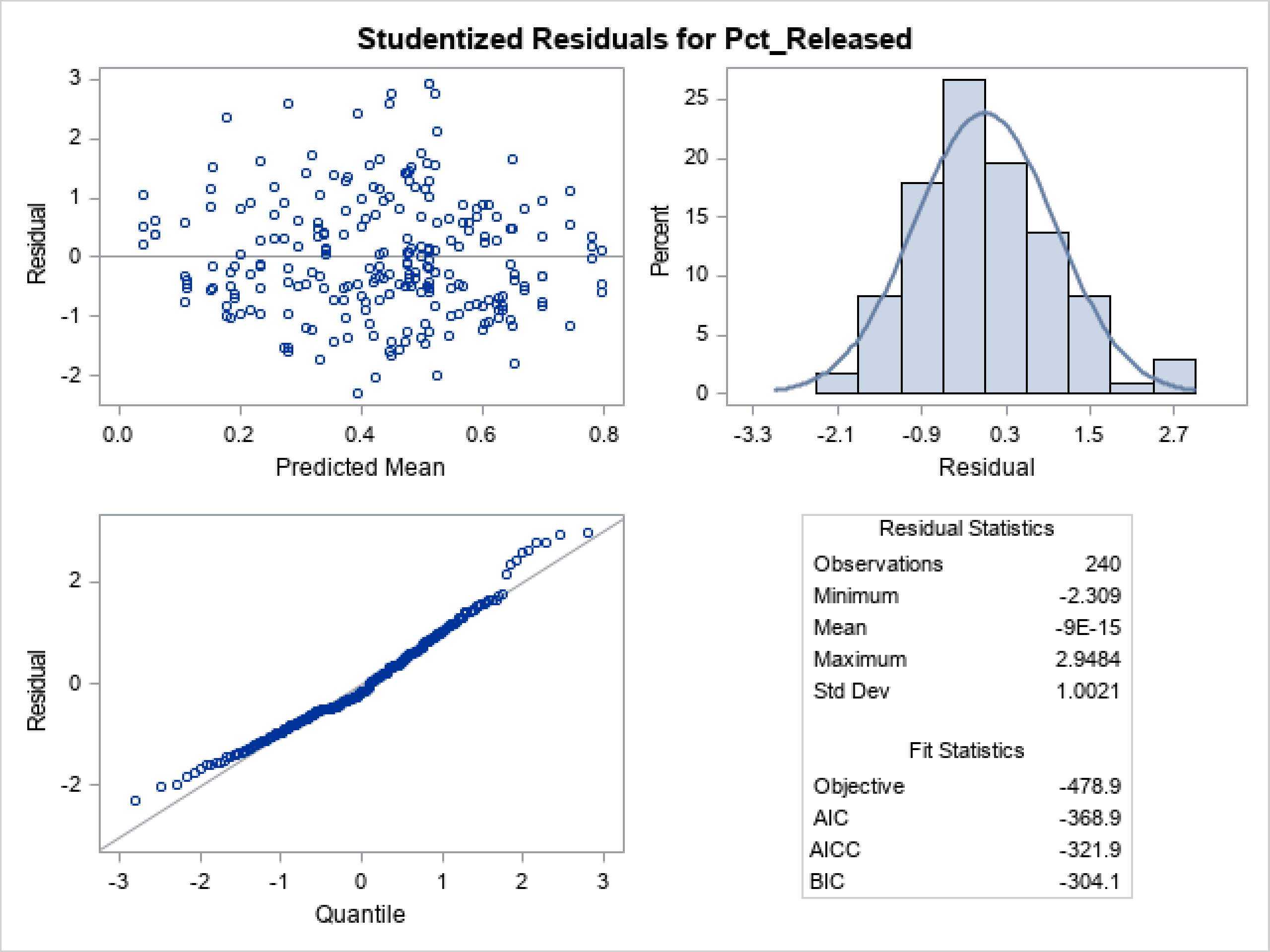
Studentized Residuals for Pct_Released
Residual
Predicted Mean
Percent
Residual
Residual
Quantile
Residual Statistics
Observations 240
Minimum -2.309
Mean -9E-15
Maximum 2.9484
Std Dev 1.0021
Fit Statistics
Objective -478.9
AIC -368.9
AICC -321.9
BIC -304.1

Pearson Residuals for Pct_Released

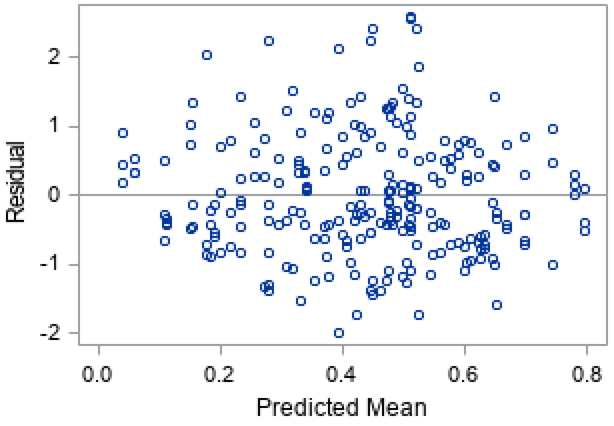

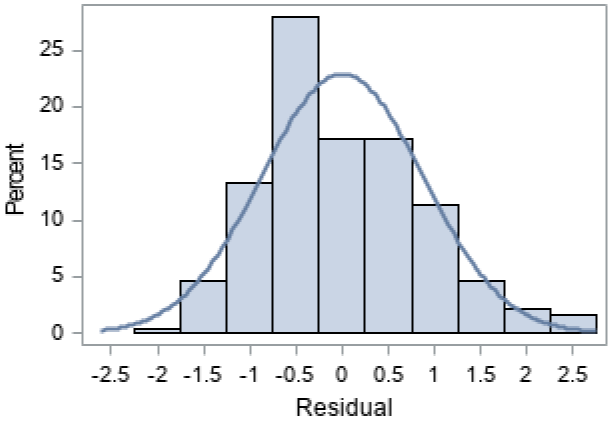

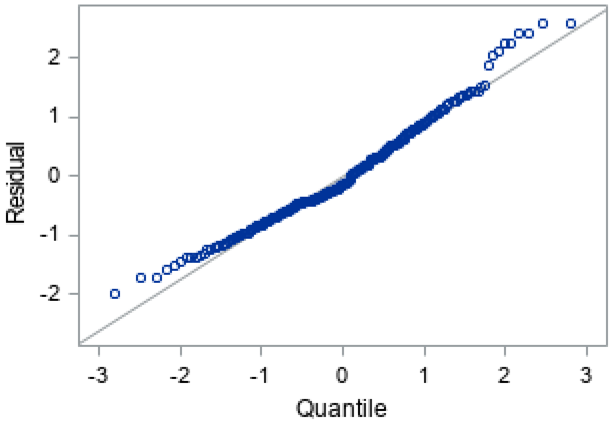

Residual Statistics	
Observations	240
Minimum	-2.002
Mean	-7E-15
Maximum	2.5851
Std Dev	0.8718

Fit Statistics	
Objective	-478.9
AIC	-368.9
AICC	-321.9
BIC	-304.1

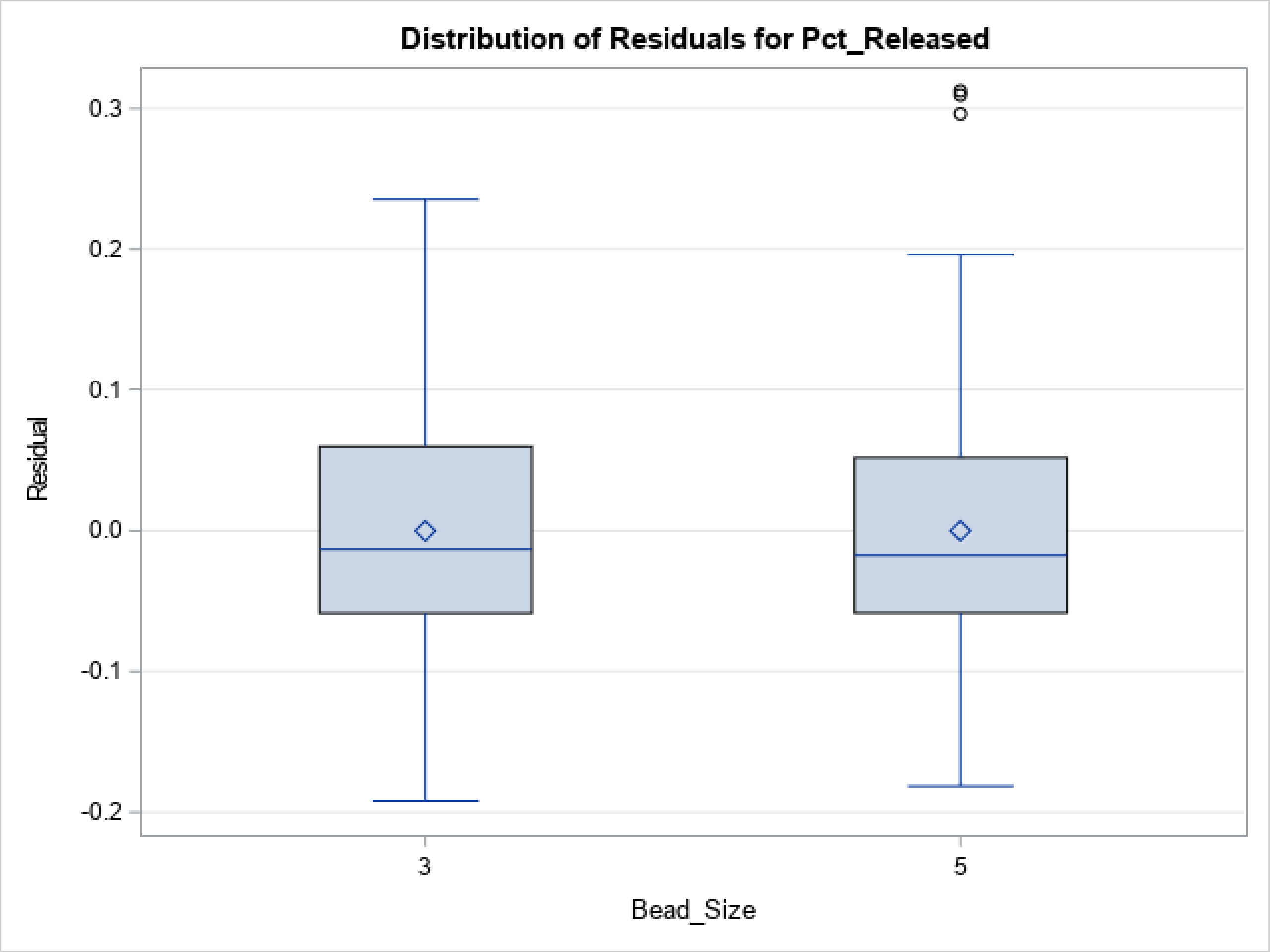
Distribution of Residuals for Pct_Released
0.3
0.2
0.1
0.0
-0.1
-0.2
Residual
3
5
Bead_Size

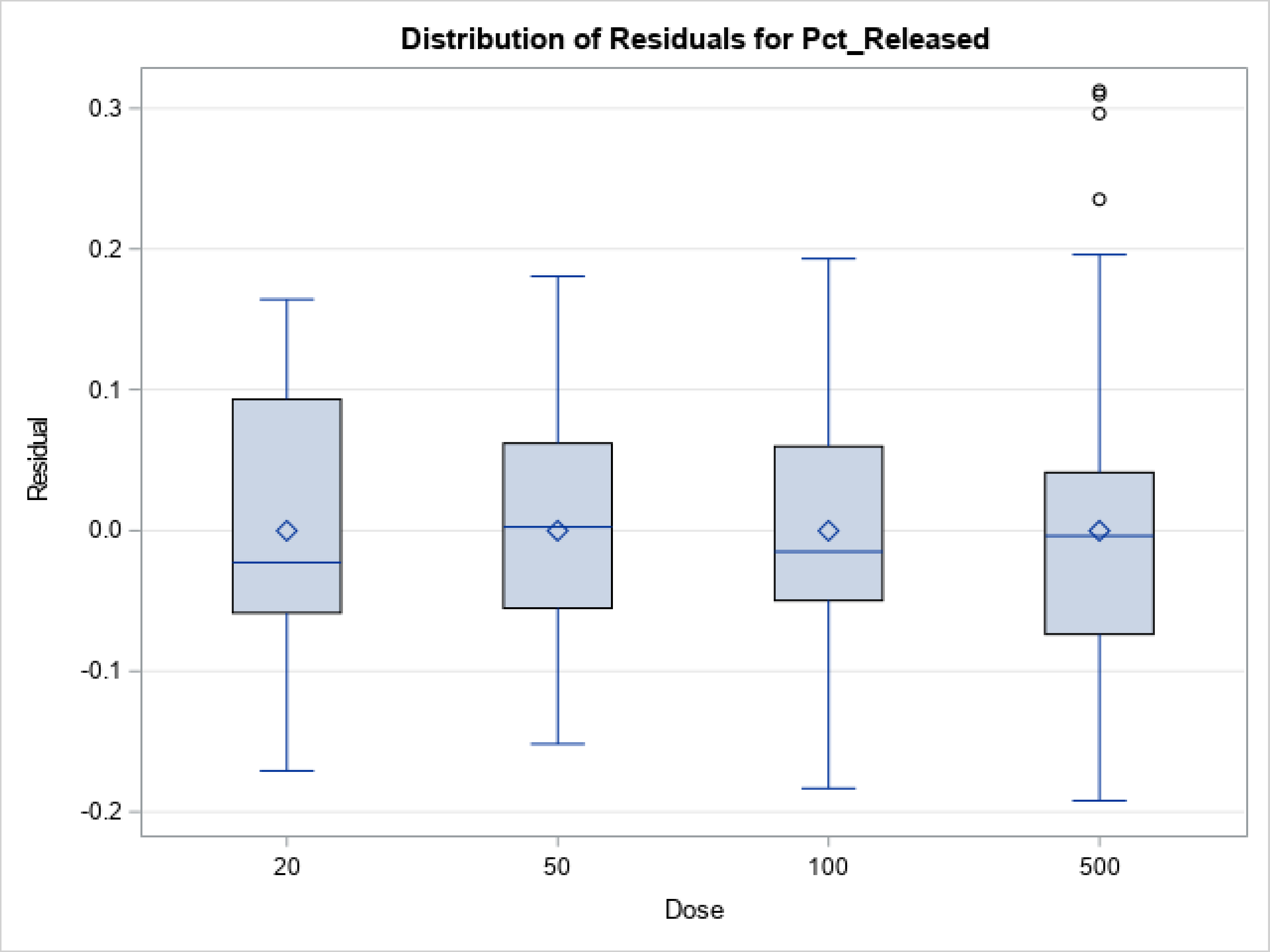
Distribution of Residuals for Pct_Released
Residual
0.3
0.2
0.1
0.0
-0.1
-0.2
20
50
100
500
Dose

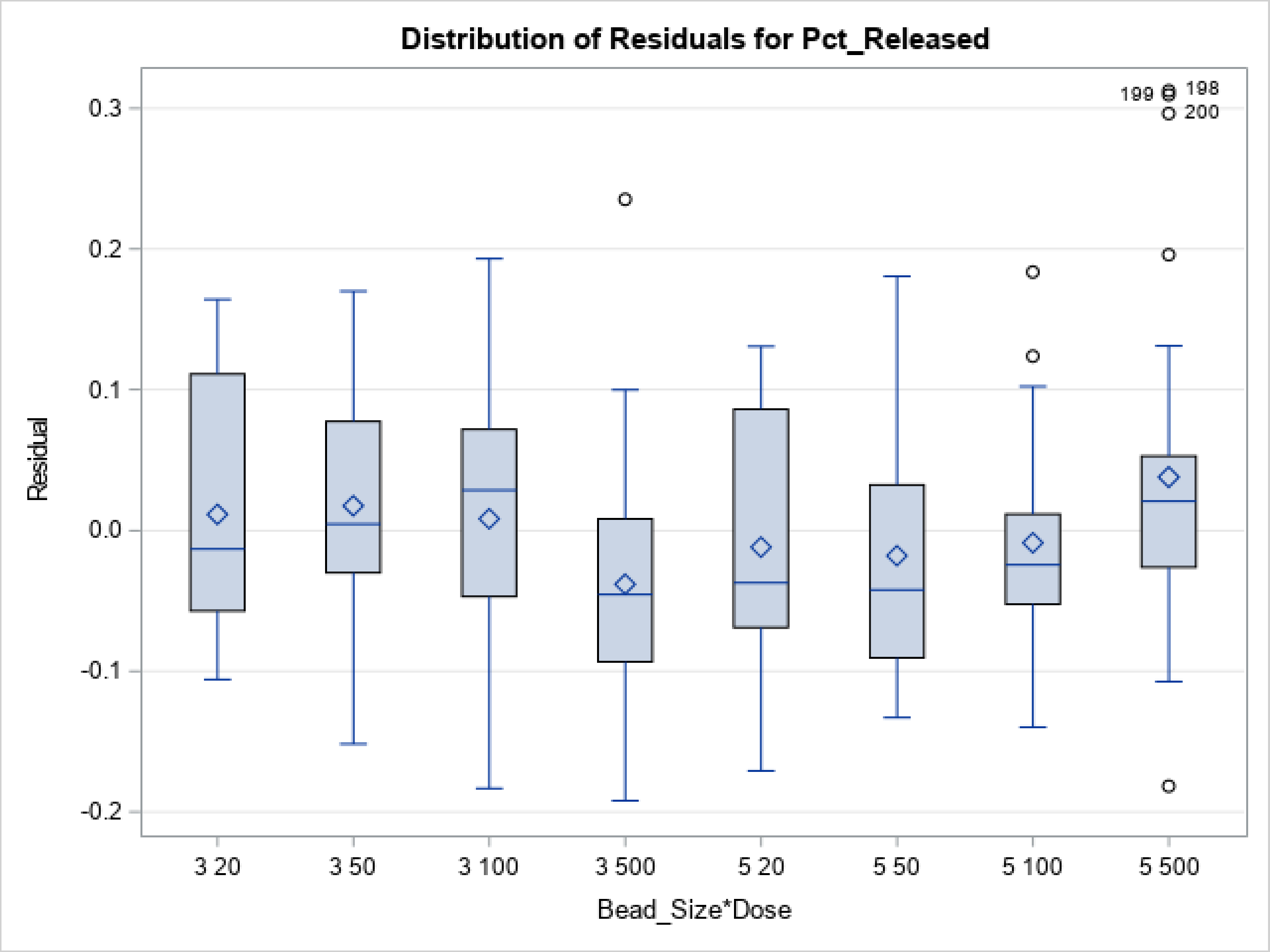
Distribution of Residuals for Pct_Released
Residual
Bead_Size*Dose
0.3
0.2
0.1
0.0
-0.1
-0.2
3 20
3 50
3 100
3 500
5 20
5 50
5 100
5 500
199
198
200

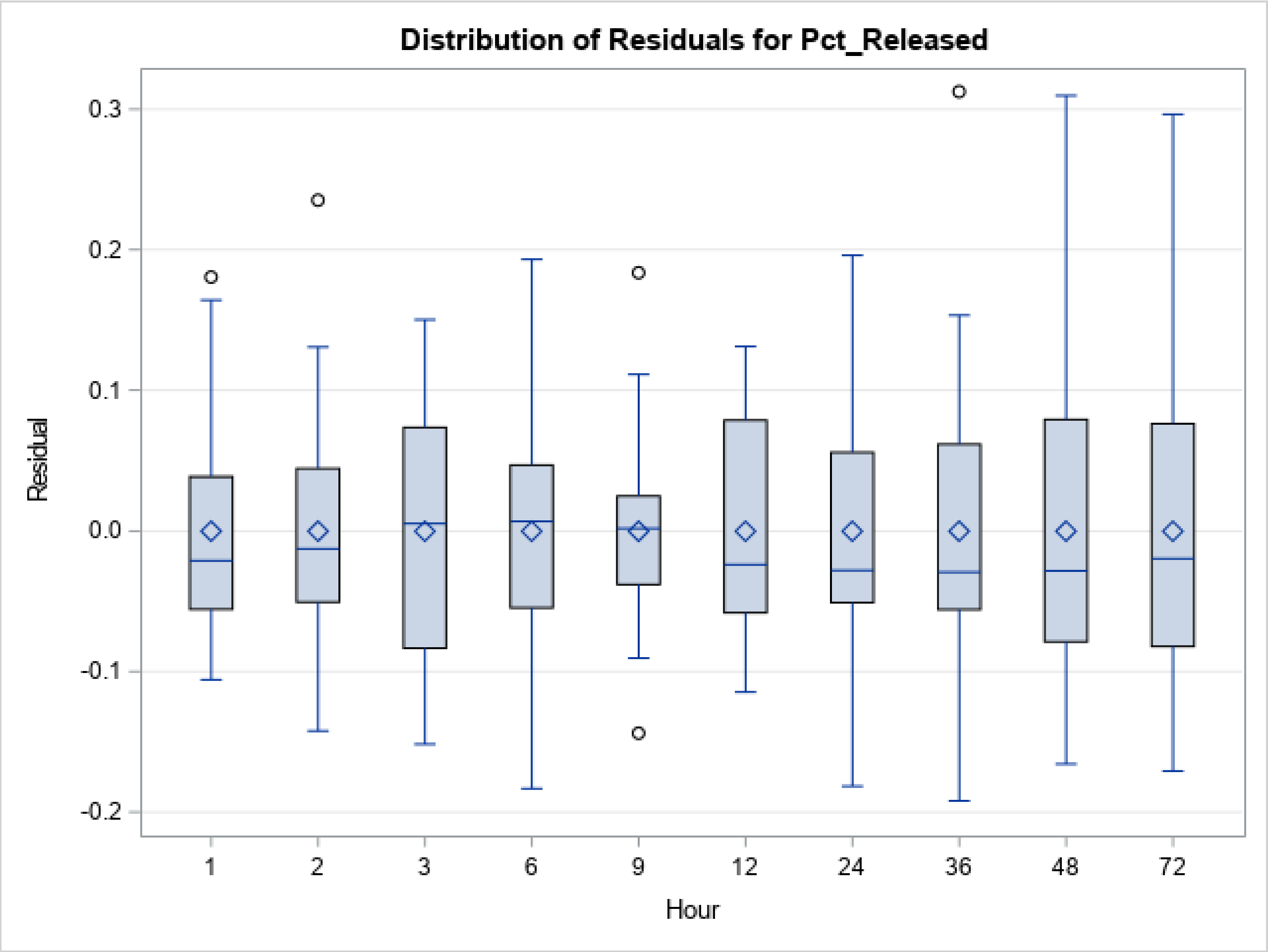
Distribution of Residuals for Pct_Released
Residual
0.3
0.2
0.1
0.0
-0.1
-0.2
1
2
3
6
9
12
24
36
48
72
Hour

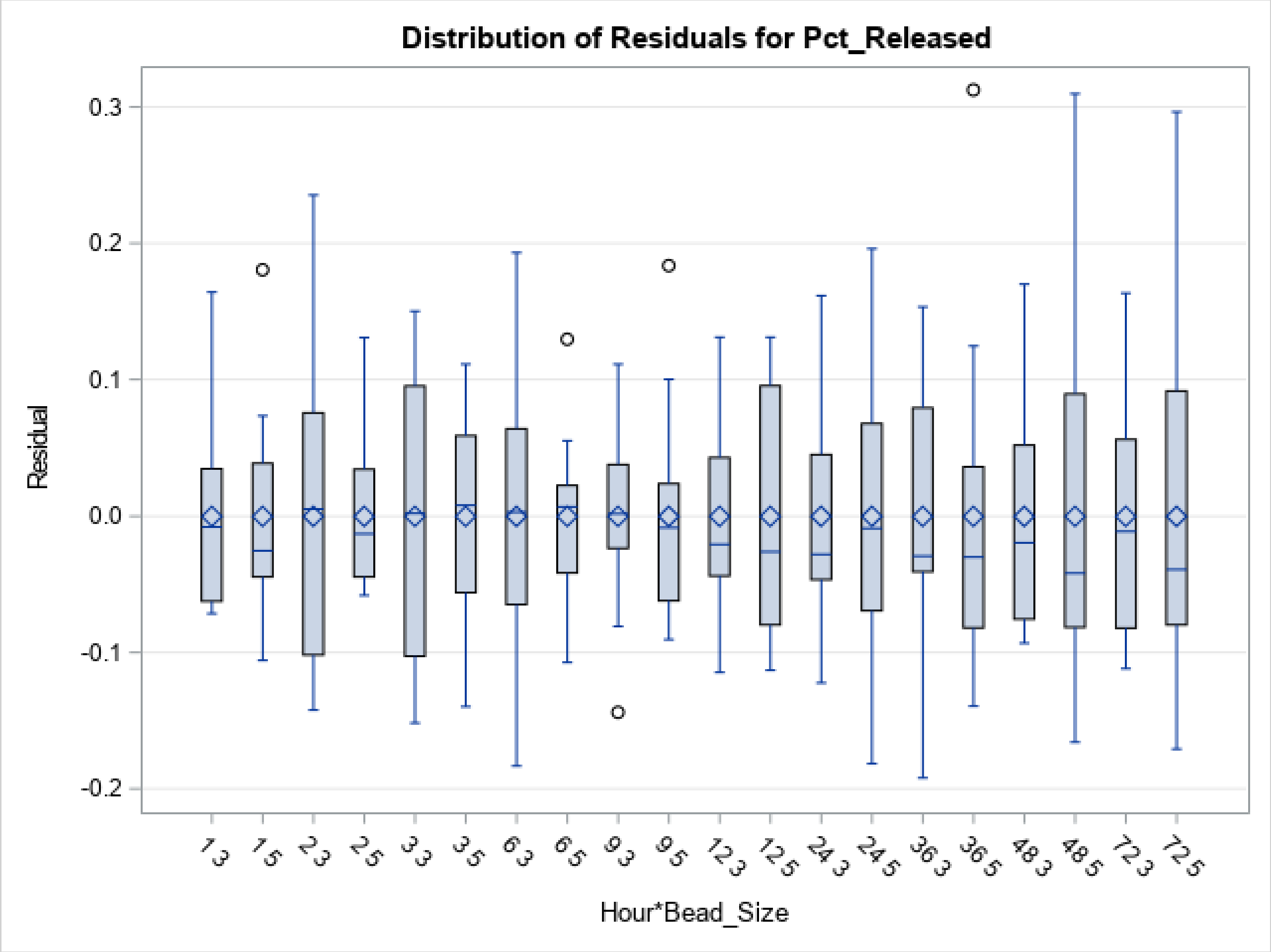
Distribution of Residuals for Pct_Released
Residual
0.3
0.2
0.1
0.0
-0.1
-0.2
1 3
1 5
2 3
2 5
3 3
3 5
6 3
6 5
9 3
9 5
12 3
12 5
24 3
24 5
36 3
36 5
48 3
48 5
72 3
72 5
Hour*Bead_Size

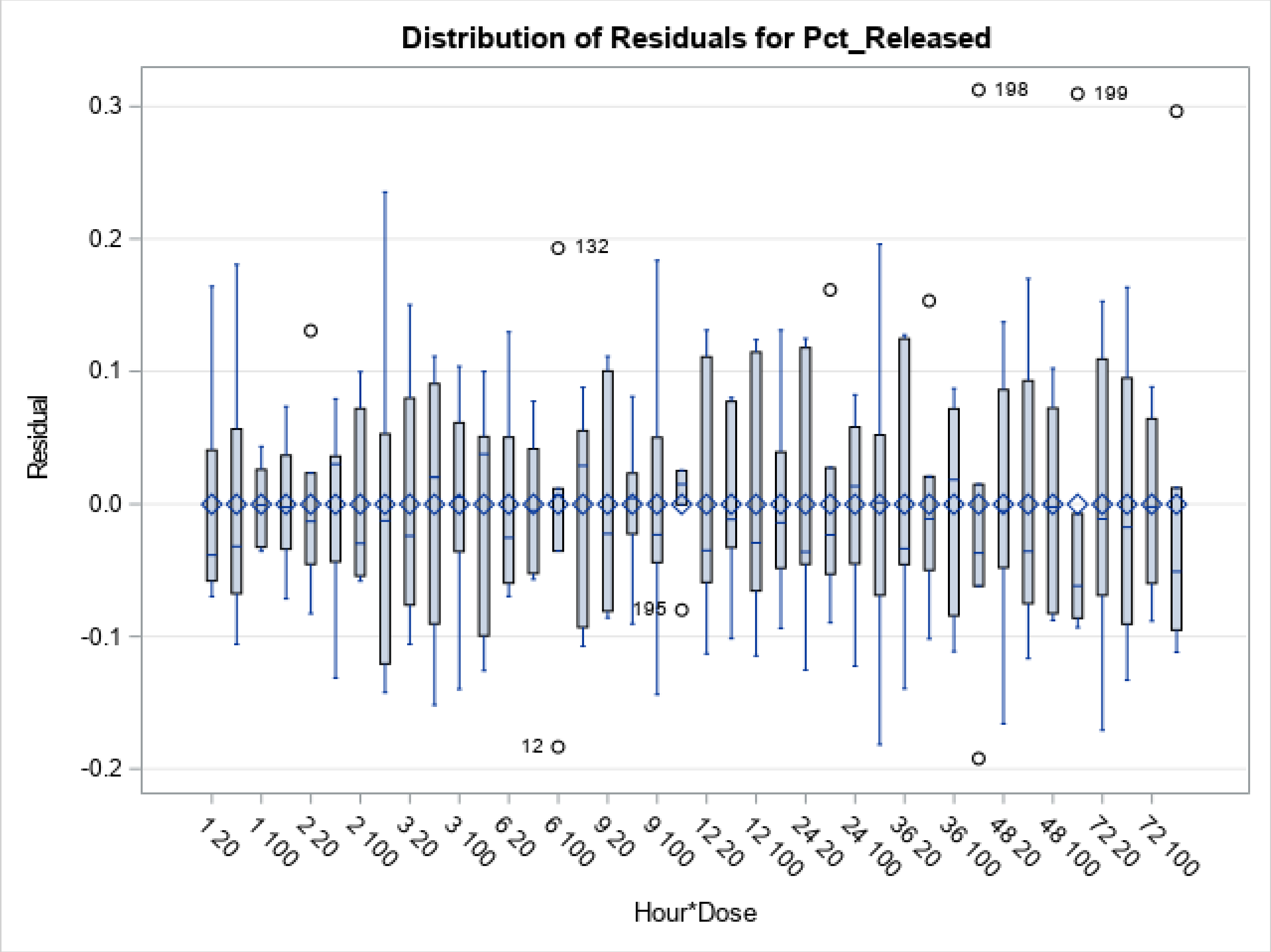
Distribution of Residuals for Pct_Released
Residual
Hour*Dose
0.3
0.2
0.1
0.0
-0.1
-0.2
198
199
132
195
12
1 20
1 100
2 20
2 100
3 20
3 100
6 20
6 100
9 20
9 100
12 20
12 100
24 20
24 100
36 20
36 100
48 20
48 100
72 20
72 100

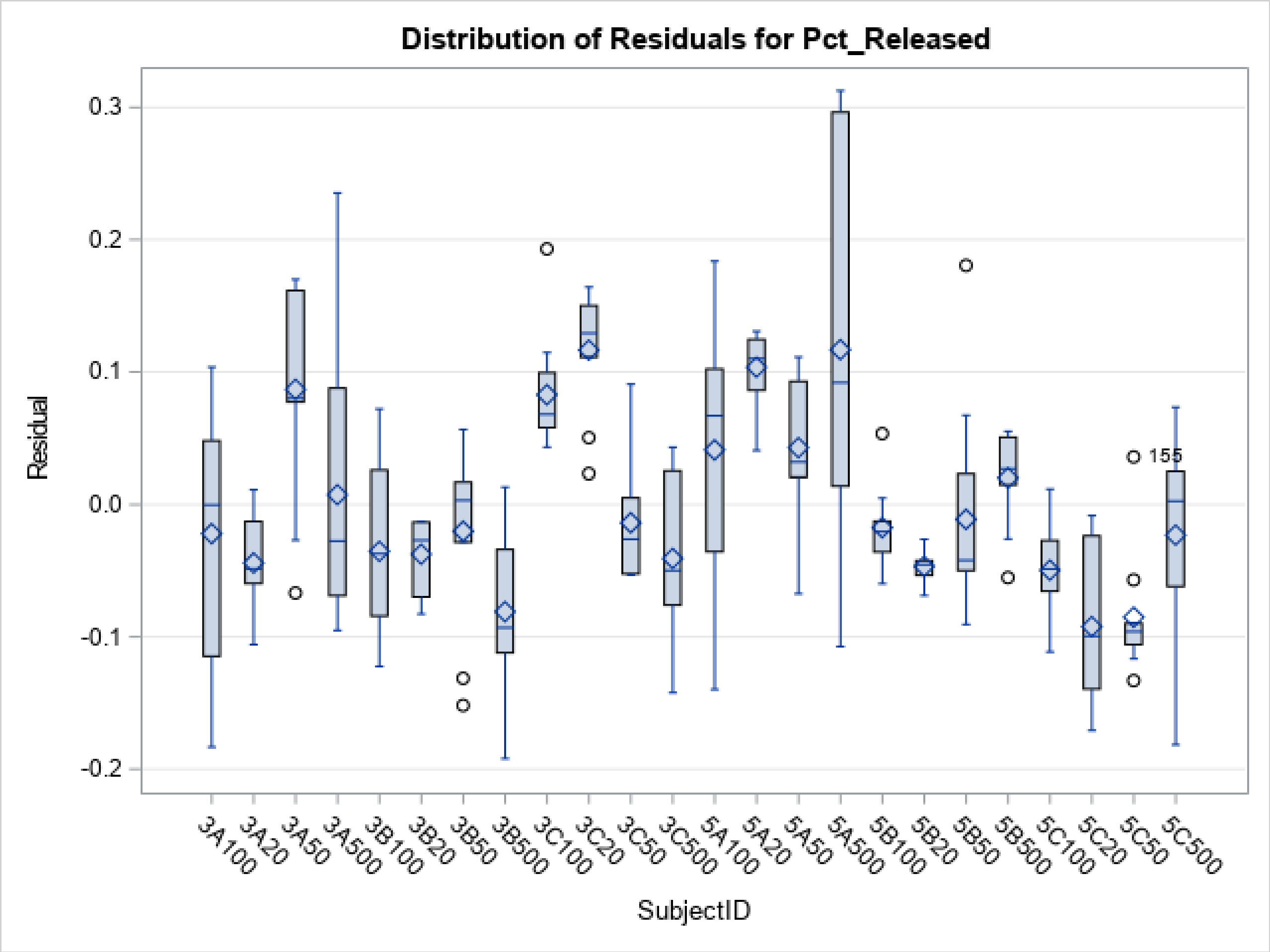
Distribution of Residuals for Pct_Released
Residual
0.3
0.2
0.1
0.0
-0.1
-0.2
155
3A100
3A20
3A50
3A500
3B100
3B20
3B50
3B500
3C100
3C20
3C50
3C500
5A100
5A20
5A50
5A500
5B100
5B20
5B50
5B500
5C100
5C20
5C50
5C500
SubjectID

The SAS System

The Mixed Procedure

Model Information	
Data Set	WORK.POWDERVLIQUID3
Dependent Variable	Pct_Released
Covariance Structure	Autoregressive Moving Average
Subject Effect	SubjectID
Estimation Method	REML
Residual Variance Method	Profile
Fixed Effects SE Method	Model-Based
Degrees of Freedom Method	Between-Within

Class Level Information

Class	Levels	Values
Hour	10	1 2 3 6 9 12 24 36 48 72
Group	3	A B C
Bead_Size	2	3 5
Type	2	Liquid Powder
SubjectID	12	3ALiquid 3APowder 3BLiquid 3BPowder 3CLiquid 3CPowder 5ALiquid 5APowder 5BLiquid 5BPowder 5CLiquid 5CPowder

Dimensions	
Covariance Parameters	3
Columns in X	99
Columns in Z	0
Subjects	12
Max Obs per Subject	10

Number of Observations	
Number of Observations Read	120
Number of Observations Used	120
Number of Observations Not Used	0

Iteration History

Iteration	Evaluations	-2 Res Log Like	Criterion

**0**	1	-117.24205467	
**1**	4	-219.55939184	.
**2**	1	-224.00429660	0.07995221
**3**	1	-227.70817113	0.00268197
**4**	1	-228.33063404	0.00068412
**5**	1	-228.47929241	0.00005805
**6**	1	-228.49081132	0.00000049
**7**	1	-228.49090300	0.00000000

Convergence criteria met.

Covariance Parameter Estimates		
**Cov Parm**	**Subject**	**Estimate**
**Rho**	SubjectID	0.9804
**Gamma**	SubjectID	0.8648
**Residual**		0.008064

Fit Statistics	
**-2 Res Log Likelihood**	-228.5
**AIC (Smaller is Better)**	-222.5
**AICC (Smaller is Better)**	-222.2
**BIC (Smaller is Better)**	-221.0

Null Model Likelihood Ratio Test		
**DF**	**Chi-Square**	**Pr > ChiSq**
2	111.25	<.0001

Information Criteria						
**Neg2LogLike**	**Parms**	**AIC**	**AICC**	**HQIC**	**BIC**	**CAIC**
-228.5	3	-222.5	-222.2	-223.0	-221.0	-218.0

Type 3 Tests of Fixed Effects				
**Effect**	**Num DF**	**Den DF**	**F Value**	**Pr > F**
**Bead_Size**	1	8	0.11	0.7484
**Type**	1	8	53.01	<.0001
**Bead_Size*Type**	1	8	3.84	0.0856
**Hour**	9	72	64.25	<.0001

**Hour*Bead_Size**	9	72	1.25	0.2782
**Hour*Type**	9	72	13.40	<.0001
**Hour*Bead_Size*Type**	9	72	1.39	0.2083

**Estimates**					
**Label**	**Estimate**	**Standard Error**	**DF**	**t Value**	**Pr > |t|**
**Powder 48**	0.4873	0.03666	72	13.29	<.0001

**Contrasts**				
**Label**	**Num DF**	**Den DF**	**F Value**	**Pr > F**
Peak comparisons	1	72	34.84	<.0001

**Least Squares Means**										
**Effect**	**Type**	**Hour**	**Estimate**	**Standard Error**	**DF**	**t Value**	**Pr > |t|**	**Alpha**	**Lower**	**Upper**
**Hour*Type**	Liquid	1	0.3767	0.03666	72	10.27	<.0001	0.05	0.3036	0.4497
**Hour*Type**	Powder	1	0.1427	0.03666	72	3.89	0.0002	0.05	0.06959	0.2157
**Hour*Type**	Liquid	2	0.6067	0.03666	72	16.55	<.0001	0.05	0.5336	0.6797
**Hour*Type**	Powder	2	0.1940	0.03666	72	5.29	<.0001	0.05	0.1209	0.2671
**Hour*Type**	Liquid	3	0.6900	0.03666	72	18.82	<.0001	0.05	0.6169	0.7631
**Hour*Type**	Powder	3	0.2680	0.03666	72	7.31	<.0001	0.05	0.1949	0.3411
**Hour*Type**	Liquid	6	0.7700	0.03666	72	21.00	<.0001	0.05	0.6969	0.8431
**Hour*Type**	Powder	6	0.3200	0.03666	72	8.73	<.0001	0.05	0.2469	0.3931
**Hour*Type**	Liquid	9	0.7900	0.03666	72	21.55	<.0001	0.05	0.7169	0.8631
**Hour*Type**	Powder	9	0.3613	0.03666	72	9.86	<.0001	0.05	0.2883	0.4344
**Hour*Type**	Liquid	12	0.7933	0.03666	72	21.64	<.0001	0.05	0.7203	0.8664
**Hour*Type**	Powder	12	0.3920	0.03666	72	10.69	<.0001	0.05	0.3189	0.4651
**Hour*Type**	Liquid	24	0.7733	0.03666	72	21.09	<.0001	0.05	0.7003	0.8464
**Hour*Type**	Powder	24	0.4277	0.03666	72	11.67	<.0001	0.05	0.3546	0.5007
**Hour*Type**	Liquid	36	0.7433	0.03666	72	20.28	<.0001	0.05	0.6703	0.8164
**Hour*Type**	Powder	36	0.4550	0.03666	72	12.41	<.0001	0.05	0.3819	0.5281
**Hour*Type**	Liquid	48	0.7333	0.03666	72	20.00	<.0001	0.05	0.6603	0.8064
**Hour*Type**	Powder	48	0.4873	0.03666	72	13.29	<.0001	0.05	0.4143	0.5604
**Hour*Type**	Liquid	72	0.6900	0.03666	72	18.82	<.0001	0.05	0.6169	0.7631
**Hour*Type**	Powder	72	0.4610	0.03666	72	12.58	<.0001	0.05	0.3879	0.5341

Tests of Effect Slices					
**Effect**	**Hour**	**Num DF**	**Den DF**	**F Value**	**Pr > F**
**Hour*Type**	1	1	72	20.37	<.0001
**Hour*Type**	2	1	72	63.36	<.0001
**Hour*Type**	3	1	72	66.26	<.0001
**Hour*Type**	6	1	72	75.34	<.0001
**Hour*Type**	9	1	72	68.36	<.0001
**Hour*Type**	12	1	72	59.92	<.0001
**Hour*Type**	24	1	72	44.45	<.0001
**Hour*Type**	36	1	72	30.93	<.0001
**Hour*Type**	48	1	72	22.51	<.0001
**Hour*Type**	72	1	72	19.51	<.0001

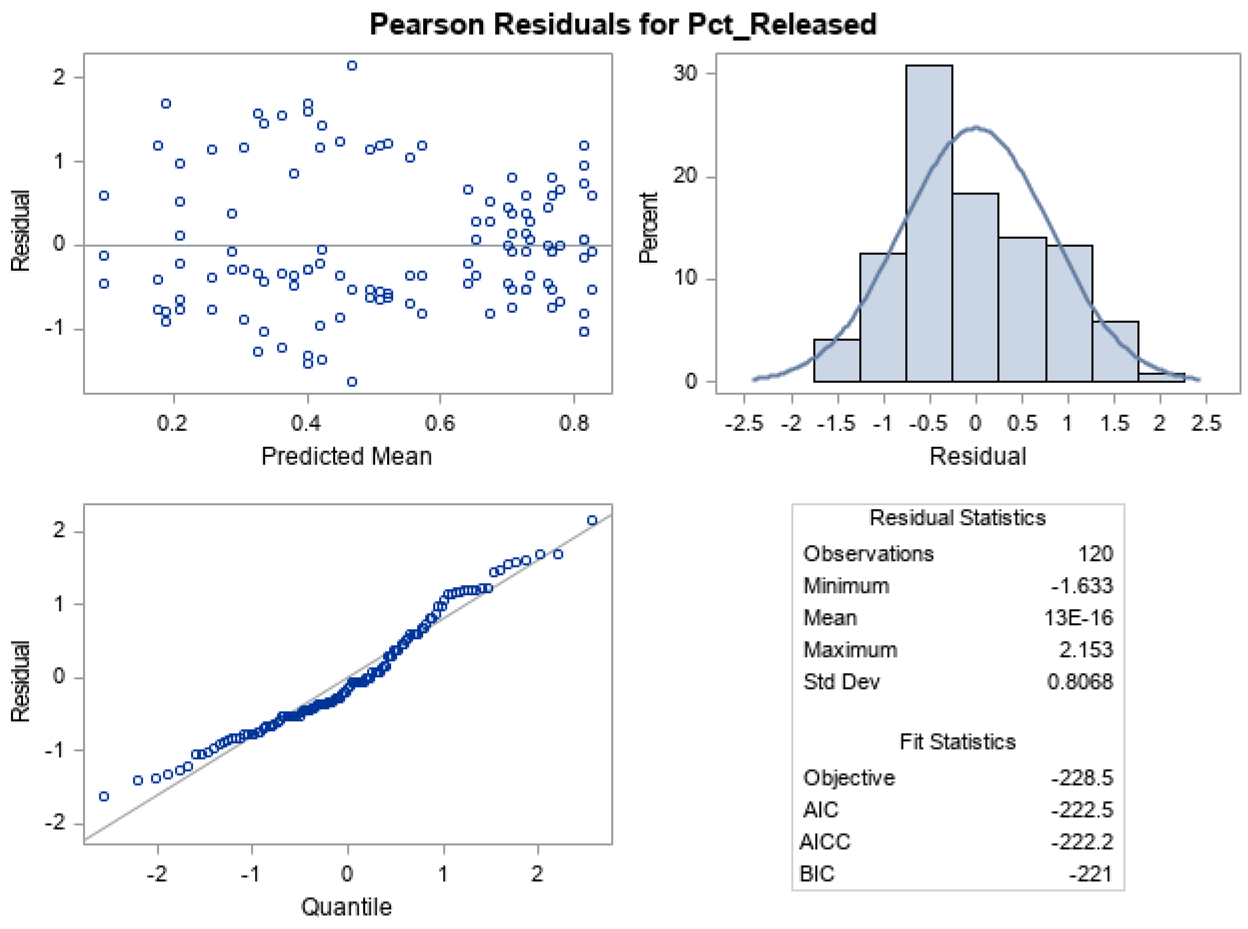

Residual Statistics	
Observations	120
Minimum	-1.633
Mean	13E-16
Maximum	2.153
Std Dev	0.8068

Fit Statistics	
Objective	-228.5
AIC	-222.5
AICC	-222.2
BIC	-221

The SAS System

The GLM Procedure

Class Level Information		
Class	Levels	Values
Bead_Size	2	3 5
Type	2	Liquid Powder

Number of Observations Read	15
Number of Observations Used	15

The SAS System

The GLM Procedure

Dependent Variable: logRateAdj

Source	DF	Sum of Squares	Mean Square	F Value	Pr > F
Model	2	15.63057149	7.81528575	62.59	<.0001
Error	12	1.49835289	0.12486274		
Corrected Total	14	17.12892438			

R-Square	Coeff Var	Root MSE	logRateAdj Mean
0.912525	-10.27439	0.353359	-3.439222

Source	DF	Type I SS	Mean Square	F Value	Pr > F
Bead_Size	1	0.01642890	0.01642890	0.13	0.7231
Type	1	15.61414259	15.61414259	125.05	<.0001

Source	DF	Type III SS	Mean Square	F Value	Pr > F
Bead_Size	1	0.11803000	0.11803000	0.95	0.3501
Type	1	15.61414259	15.61414259	125.05	<.0001

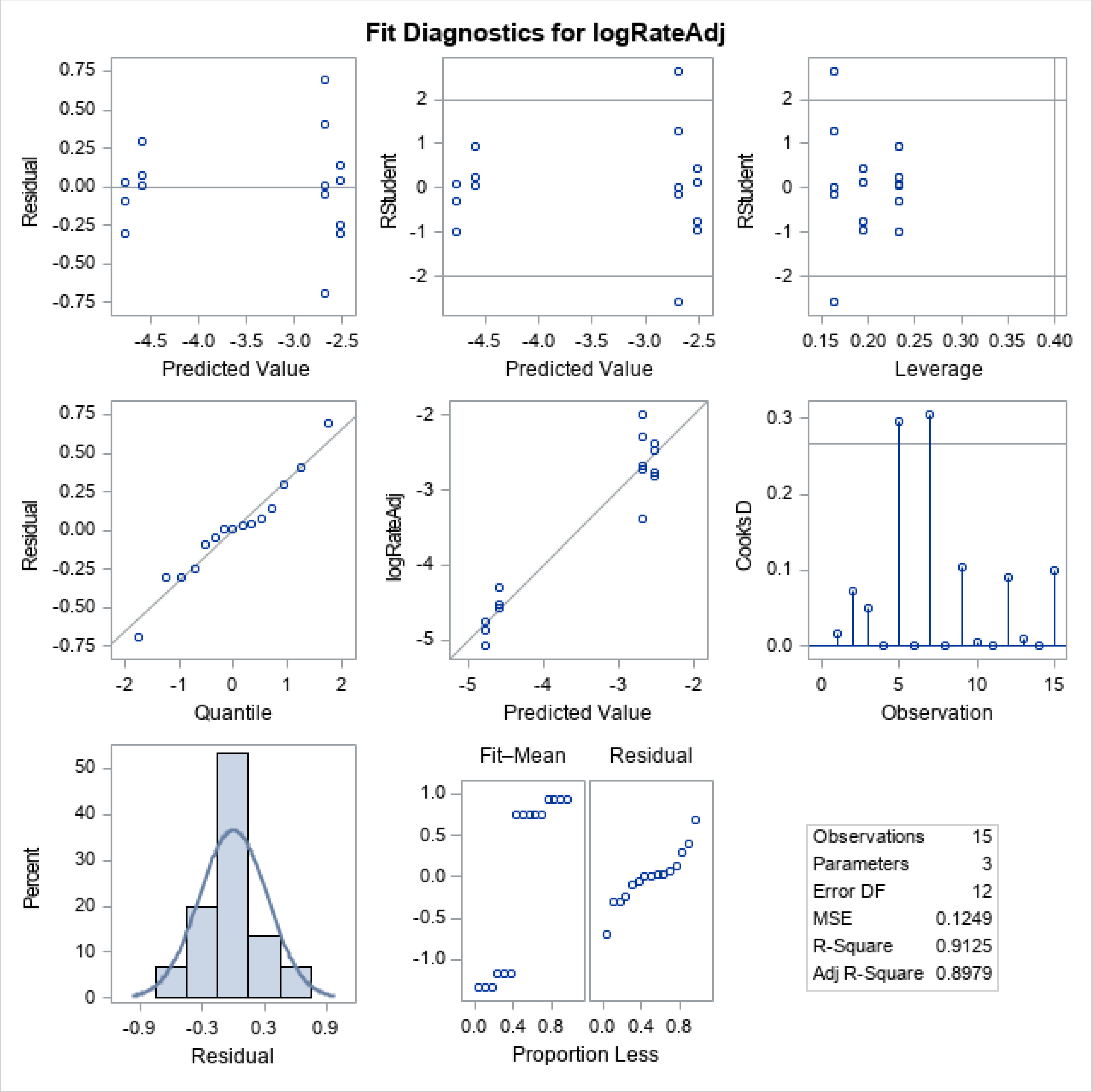
Fit Diagnostics for logRateAdj
Residual
Predicted Value
RStudent
Predicted Value
RStudent
Leverage
Residual
Quantile
logRateAdj
Predicted Value
Cook's D
Observation
Percent
Residual
Fit–Mean
Residual
Proportion Less
Observations 15
Parameters 3
Error DF 12
MSE 0.1249
R-Square 0.9125
Adj R-Square 0.8979

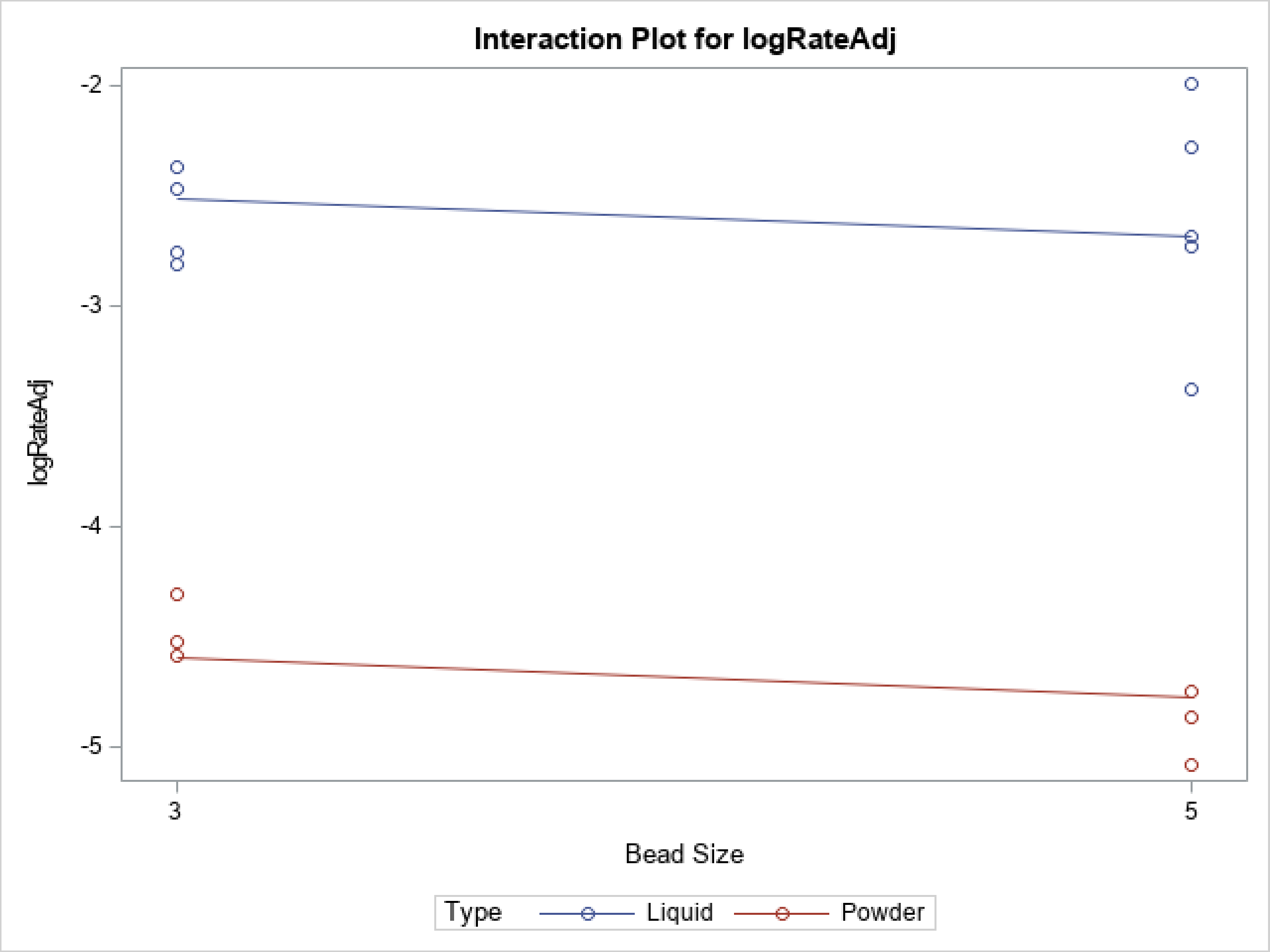
Interaction Plot for logRateAdj
-2
-3
-4
-5
logRateAdj
3
5
Bead Size
Type
Liquid
Powder

The SAS System

The GLM Procedure
Least Squares Means
Adjustment for Multiple Comparisons: Tukey-Kramer

Type	logRateAdj LSMEAN	Standard Error	H0:LSMEAN=0	H0:LSMean1=LSMean2
			Pr > |t|	Pr > |t|
Liquid	-2.59900052	0.11822509	<.0001	<.0001
Powder	-4.68471596	0.14425830	<.0001	

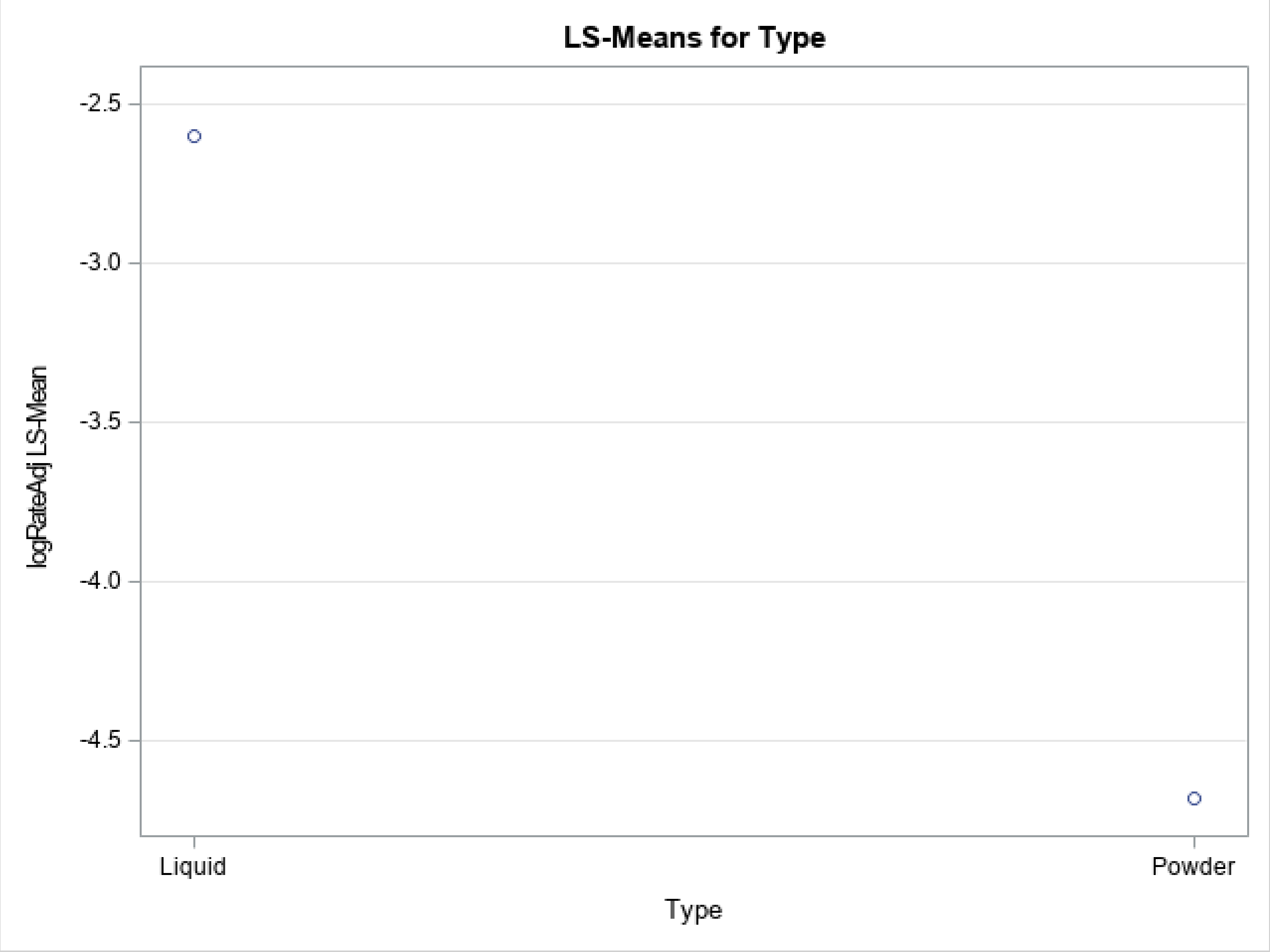

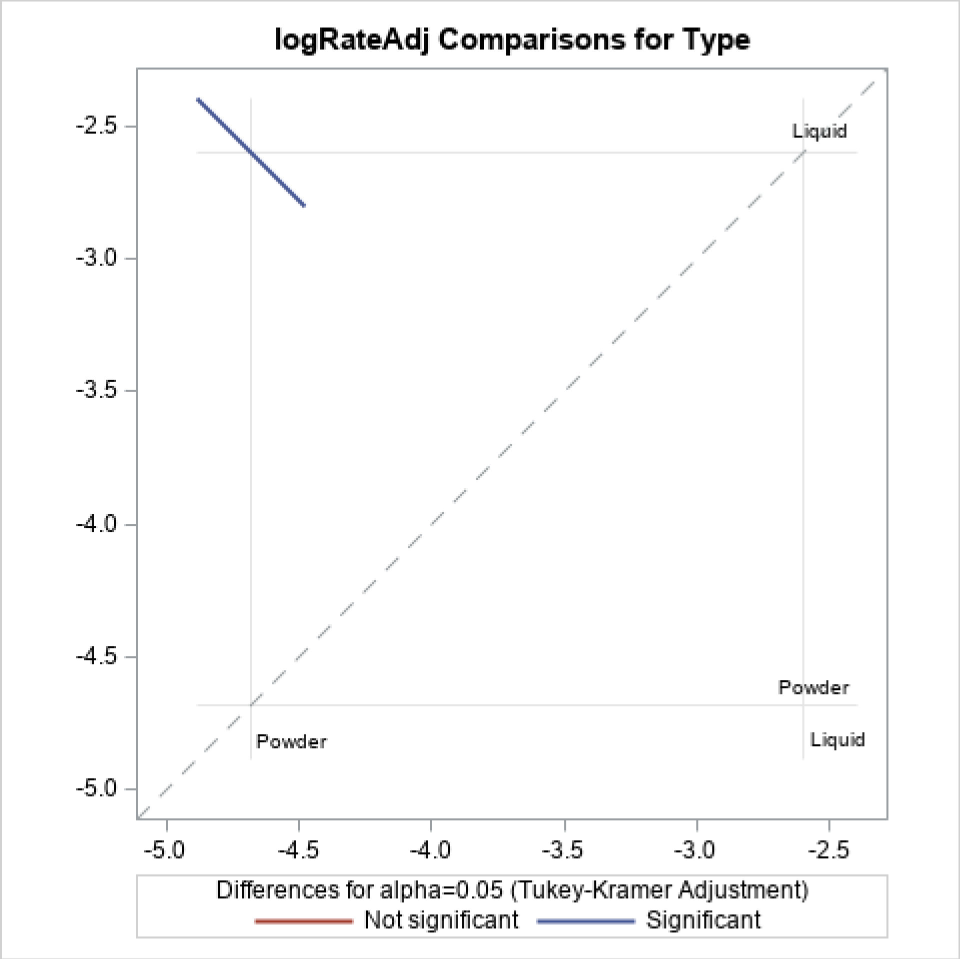
logRateAdj Comparisons for Type
-2.5
-3.0
-3.5
-4.0
-4.5
-5.0
Liquid
Powder
Powder
Liquid
-5.0 -4.5 -4.0 -3.5 -3.0 -2.5
Differences for alpha=0.05 (Tukey-Kramer Adjustment)
Not significant
Significant

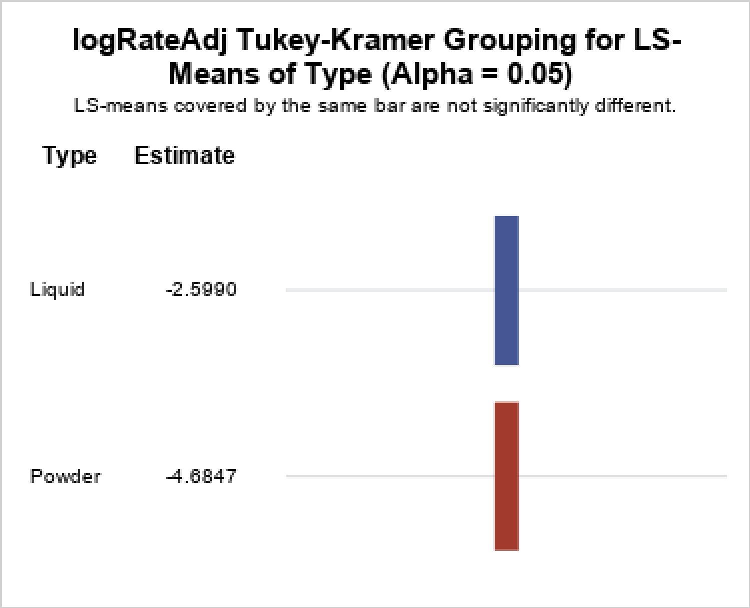
logRateAdj Tukey-Kramer Grouping for LS-Means of Type (Alpha = 0.05)
LS-means covered by the same bar are not significantly different.
Type
Estimate
Liquid
-2.5990
Powder
-4.6847
