# Supplementary material for: Evaluation of bioabsorbable calcium sulfate hemihydrate beads for local delivery of carboplatin
Source: PLoS One. 2020 Nov 5;15(11):e0241718. doi: 10.1371/journal.pone.0241718 (PMC7644016; doi:10.1371/journal.pone.0241718)
Supplement: S1 Statistics code — (DOCX) [file pone.0241718.s003.docx]

/************************************************************/

/* SAS Code to run all models in paper

Carboplatic elution from carboplatin-impregnated

calcium sulfate beads

*/

/************************************************************/

* Read in raw data;

libname Files XLSX 'C:/Users/eghohmei/Documents/Consulting 2020 Summer/Marine CVM/Clean Data.xlsx';

*Separate raw data into the two studies;

**data** PowderStudy;

set Files.SHEET1;

where Type = 'Powder';

**run**;

**data** PowdervLiquid;

set Files.SHEET1;

where Sample = **20**;

**run**;

* Now clean and organize the Study 1 data;

**data** work.PowderStudy2;

set work.PowderStudy (rename=(Sample = Dose));

label Dose = 'Dose';

if ug_ml = **.** then ug_ml = **0**;

check = Adj_ug_ml / ug_ml;

*Now calculate the Amount Released;

Amt_Released_mg = ug_ml * **200** / **1000**;

if Dose = **0** then Pct_Released = **.**;

else Pct_Released = Amt_Released_mg / (Dose/**2**); *Note: Dose is also in mg;

drop Adj_ug_ml; *The adjusted mg are already doubled;

**run**;

* Plot the data;

**proc** **sgpanel** data= PowderStudy2;

panelby Group Bead_Size /columns=**2**;

series x = Hour y = ug_ml /group=Dose;

**run**;

**proc** **sgpanel** data= PowderStudy2;

panelby Group Bead_Size /columns=**2**;

series x = Hour y = Amt_Released_mg /group=Dose;

**run**;

/************************************************************/

*Create Table 1;

**proc** **means** data=PowderStudy2 n mean stddev maxdec=**2**;

class Dose Bead_Size Hour;

var ug_ml;

output out=PowderStudyMeans;

**run**;

**data** PowderStudyMeans2;

set PowderStudyMeans (where=(_TYPE_ = **5** or _TYPE_ = **7**));

if _STAT_ in ('MIN','MAX') then delete;

**run**;

**proc** **sort** data=PowderStudyMeans2;

by Dose Bead_Size Hour;

**run**;

**proc** **transpose** data=PowderStudyMeans2 out=Table1Stats;

by Dose Bead_Size Hour;

id _STAT_;

var ug_ml;

**run**;

**proc** **sql**;

create table Table1final as

select Dose, Hour, Bead_Size, N, STD, max(MEAN) as max_MEAN

from Table1Stats

group by Dose, Bead_Size

having MEAN = max_MEAN

;

**quit**;

**proc** **means** data=PowderStudy2 n mean stddev maxdec=**2**;

class Dose Bead_Size Hour;

var Pct_Released;

output out=PowderStudyPctMeans;

**run**;

**data** PowderStudyPctMeans2;

set PowderStudyPctMeans (where=(_TYPE_ = **5** or _TYPE_ = **7**));

if _STAT_ in ('MIN','MAX') then delete;

**run**;

**proc** **sort** data=PowderStudyPctMeans2;

by Dose Bead_Size Hour;

**run**;

**proc** **transpose** data=PowderStudyPctMeans2 out=Table1PctStats;

by Dose Bead_Size Hour;

id _STAT_;

var Pct_Released;

**run**;

**data** Table1final; set Table1final;

Amt_Released_mg = max_MEAN * **200** / **1000**;

if Dose ne **0** then Pct_Released = Amt_Released_mg / (Dose/**2**);

else Pct_Released = **0**;

**run**;

**proc** **print** data=Table1final;

var Dose Bead_Size max_MEAN STD Pct_Released;

**run**;

/************************************************************/

*Create Table 2;

**data** work.PowdervLiquid2;

set work.PowdervLiquid (rename=(Sample = Dose));

label Dose = 'Dose';

if ug_ml = **.** then ug_ml = **0**;

*Now calculate the Amount Released;

Amt_Released_mg = ug_ml * **200** / **1000**;

Pct_Released = Amt_Released_mg / (Dose/**2**); *Note: Dose is also in mg;

drop Adj_ug_ml; *The adjusted mg are all missing here;

**run**;

* Plot the data;

**proc** **sgpanel** data= PowdervLiquid2;

panelby Bead_Size Type /columns=**2**;

series x = Hour y = ug_ml /group=Group;

**run**;

**proc** **sgpanel** data= PowdervLiquid2;

panelby Bead_Size /columns=**2**;

series x = Hour y = Amt_Released_mg /group=Type ;

**run**;

/************************************************************/

*Recreate Table 2;

**proc** **means** data=PowdervLiquid2 n mean stddev maxdec=**2**;

class Type Bead_Size Hour;

var ug_ml;

output out=PowdervLiquidMeans;

**run**;

**data** PowdervLiquidMeans2;

set PowdervLiquidMeans (where=(_TYPE_ = **5** or _TYPE_ = **7**));

if _STAT_ in ('MIN','MAX') then delete;

**run**;

**proc** **sort** data=PowdervLiquidMeans2;

by Type Bead_Size Hour;

**run**;

**proc** **transpose** data=PowdervLiquidMeans2 out=Table2Stats;

by Type Bead_Size Hour;

id _STAT_;

var ug_ml;

**run**;

**proc** **sql**;

create table Table2final as

select Type, Hour, Bead_Size, N, STD, max(MEAN) as max_MEAN

from Table2Stats

group by Type, Bead_Size

having MEAN = max_MEAN

;

**quit**;

**data** Table2final; set Table2final;

Amt_Released_mg = max_MEAN * **200** / **1000**;

Pct_Released = Amt_Released_mg / (**20**/**2**); *Dose = 20;

**run**;

**proc** **print** data=Table2final;

var Type Bead_Size max_MEAN STD Pct_Released;

**run**;

*Plot the means by group;

**proc** **means** data=PowdervLiquid2 n mean stddev maxdec=**2**;

class Type Bead_Size Hour;

var Pct_Released;

output out=PowdervLiquidPctMeans;

**run**;

**data** PowdervLiquidPctMeans2;

set PowdervLiquidPctMeans (where=(_TYPE_ = **5** or _TYPE_ = **7**));

if _STAT_ in ('MIN','MAX') then delete;

**run**;

**proc** **sort** data=PowdervLiquidPctMeans2;

by Type Bead_Size Hour;

**run**;

**proc** **transpose** data=PowdervLiquidPctMeans2 out=Table2PctStats;

by Type Bead_Size Hour;

id _STAT_;

var Pct_Released;

**run**;

**proc** **sgpanel** data= Table2Stats;

panelby Bead_Size /columns=**2**;

series x = Hour y = MEAN /group=Type ;

**run**;

/************************************************************/

* Study 1 data analysis;

*Time to peak;

*Create the dataset;

* Step 1: Make a unique ID;

**data** PowderStudy3; set PowderStudy2;

logHour = log(Hour+**.5**);

SubjectID = strip(Bead_Size) || strip(Group) || strip(Dose);

**run**;

* Plot the data;

**proc** **sgpanel** data= PowderStudy3;

panelby Group Bead_Size /columns=**2**;

series x = logHour y = ug_ml /group=Dose;

**run**;

**proc** **sgpanel** data= PowderStudy3;

panelby Group Bead_Size /columns=**2**;

series x = logHour y = Amt_Released_mg /group=Dose;

**run**;

**proc** **sgpanel** data= PowderStudy3;

panelby Group Bead_Size /columns=**2**;

series x = Hour y = Pct_Released /group=Dose;

**run**;

**proc** **sgpanel** data= PowderStudy3;

panelby Dose Bead_Size /columns=**2**;

series x = Hour y = Pct_Released /group=Group;

**run**;

* Format the data for analysis;

**proc** **sql**;

create table TimetoPeakStudy1 as

select Type, Hour, SubjectID, Bead_Size, Dose, Pct_Released, max(Amt_Released_mg) as max_Amt

from PowderStudy3

group by Type, Bead_Size, SubjectID

having Amt_Released_mg = max_Amt

;

**quit**;

**data** TimetoPeakStudy1_2; set TimetoPeakStudy1;

where Dose ne **0**;

**run**;

*Time to peak;

**proc** **glm** data=TimetoPeakStudy1_2 plots=diagnostics;

class Bead_Size Dose;

model Hour = Bead_Size| Dose;

lsmeans Bead_Size Dose /pdiff=all stderr;

**run**;

*Peak percentage;

**proc** **glm** data=TimetoPeakStudy1_2 plots=diagnostics;

class Bead_Size Dose;

model Pct_Released = Bead_Size Dose;

* lsmeans Dose /pdiff=all stderr lines;

**run**;

*Percentage at 72 hours;

**proc** **glm** data=PowderStudy3 plots=diagnostics;

where hour = **72**;

class Bead_Size Dose;

model Pct_Released = Bead_Size Dose;

lsmeans Dose /pdiff=all stderr lines;

**run**;

*Rate to peak;

**data** TimetoPeakStudy1_3;

set TimetoPeakStudy1_2;

RateAdj = Pct_Released/Hour;

logRateAdj = log(RateAdj);

**run**;

**proc** **glm** data=TimetoPeakStudy1_3 plots=diagnostics;

where Dose ne **0**;

class Bead_Size Dose;

model logRateAdj = Bead_Size Dose;

lsmeans Dose Bead_Size/pdiff=all stderr lines;

**run**;

**proc** **sgplot** data=TimetoPeakStudy1_3;

histogram RateAdj;

**run**;

**proc** **mixed** data=PowderStudy3 plots=all IC;

where Dose ne **0** and Hour > **0**;

class Hour Group Bead_Size Dose SubjectID;

model Pct_Released = Bead_Size | Dose | Hour @**2**;

repeated Hour/subject=SubjectID type=UN;

lsmeans Bead_Size*Hour/slice=hour;

lsmeans Dose*Hour /slice = hour;

**run**;

*Liquid v Powder study;

* Step 1: Make a unique ID;

**data** PowdervLiquid3; set PowdervLiquid2;

SubjectID = strip(Bead_Size) || strip(Group) || strip(Type);

**run**;

ods output lsmeans = FormulationMeans;

**proc** **mixed** data=PowdervLiquid3 plots=pearsonpanel IC;

where Hour > **0**;

class Hour Group Bead_Size Type SubjectID;

model Pct_Released = Bead_Size | Type | Hour @**3**;

repeated Hour/subject=SubjectID type=ARMA(**1**,**1**);

lsmeans Type*Hour/ slice=hour cl;

estimate 'Powder 48' Intercept **1**

Type **0** **1**

Hour **0** **0** **0** **0** **0** **0** **0** **0** **1** **0**

Type*Hour **0** **0** **0** **0** **0** **0** **0** **0** **0** **0**

**0** **0** **0** **0** **0** **0** **0** **1** **0** **0**;

contrast 'Peak comparisons' Type **1** -**1**

Hour **0** **0** **0** **0** **0** **1** **0** **0** -**1** **0**

Type*Hour **0** **0** **0** **0** **0** **0** **0** **0** **0** **0**

**1** **0** **0** **0** **0** **0** **0** -**1** **0** **0**;

**run**;

**proc** **sgplot** data=FormulationMeans;

series x=Hour y=Estimate /group=Type;

band x=Hour lower=lower upper=upper/group=Type transparency = **0.5**;

**run**;

*Time to peak Study 2;

**proc** **sql**;

create table TimetoPeakStudy2 as

select Type, Hour, SubjectID, Bead_Size, Dose, Pct_Released, max(Amt_Released_mg) as max_Amt

from PowdervLiquid3

group by Type, Bead_Size, SubjectID

having Amt_Released_mg = max_Amt

;

**quit**;

**data** TimetoPeakStudy2_2; set TimetoPeakStudy2;

where Dose ne **0**;

RateAdj = Pct_Released/Hour;

logRateAdj = log(RateAdj);

**run**;

**proc** **glm** data=TimetoPeakStudy2_2 plots=diagnostics;

class Bead_Size Type;

model logRateAdj = Bead_Size Type;

lsmeans Type/pdiff=all stderr lines;

**run**;
